# Synthesis and Structural Analysis of an Emissive Colloidal Argyrodite Nanocrystal: Canfieldite Ag_8_SnS_6_

**DOI:** 10.1021/jacs.5c09495

**Published:** 2025-07-30

**Authors:** Francisco Yarur Villanueva, Victor Quezada Novoa, Pascal Rusch, Stefano Toso, Maxwell W. Terban, Yurii P. Ivanov, Joaquin Carlos Chu, Maxine J. Kirshenbaum, Ehsan Nikbin, Maria J. Gendron Romero, Mirko Prato, Giorgio Divitini, Jane Y. Howe, Mark W. B. Wilson, Liberato Manna

**Affiliations:** † 121451 Istituto Italiano di Tecnologia, Via Morego 30, 16163 Genova, Italy; ‡ Department of Chemistry, 7938 University of Toronto, Toronto, Ontario M5S 3H6, Canada; § Department of Chemistry and Biochemistry and Centre for NanoScience Research, 5618 Concordia University, 7141 Sherbrooke Street West, Montréal, Quebec H4B 1R6, Canada; ∥ Momentum Transfer GmbH, Luruper Hauptstraße 1, 22547 Hamburg, Germany; ⊥ University of Toronto, Department of Materials Science and Engineering, Toronto, Ontario M5S3E4, Canada

## Abstract

We resolve a phase identification controversy in the Ag–Sn–S material system by unraveling the polymorphic structure of nanocrystals within the argyrodite material family. Argyrodites are a class of superionic materials used in their bulk form for applications in solid-state batteries and thermoelectrics, where their advantageous properties relate to their polymorphism. However, despite their well-studied bulk applications, the limited exploration at the nanoscale has left considerable potential for the discovery of emerging properties due to size effects. Further, phase identification presents a prominent challenge to the study of polymorphs in superionic conductors and related materials. In this work, we synthesize canfieldite-like (Ag_8_SnS_6_) nanocrystals to understand their formation and structural behavior at the nanoscale. We observe the emergence of emissive, metastable, cluster-like species. Then, high-resolution transmission electron microscopy reveals indistinguishable polymorphs of canfieldite due to identical heavy-atom frameworks. However, using synchrotron X-ray total scattering for pair distribution function analysis, we uncover structural distortions, showing a pseudo-orthorhombic configuration that likely gives rise to the red emission. Further, we investigate the optical properties and structure of Ag_8_SnS_6_ nanocrystals upon the addition of Zn^2+^, the cation of interest in the canfieldite vs pirquitasite (Ag_2_ZnSnS_4_) phase identification controversy. We show that Zn^2+^ is incorporated in the canfieldite-like structure through the replacement of Ag^+^, boosting the emission. Our results solve a standing phase identification challenge and uncover fundamental insights for the synthesis and structure of canfieldite nanocrystals, laying the ground for the exploration of other argyrodite materials with emerging properties at the nanoscale.

## Graphical abstract

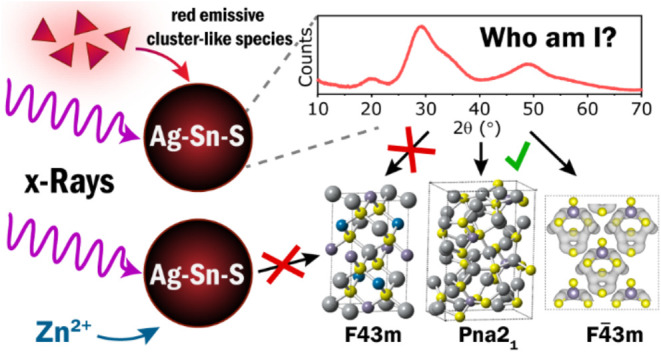

## 1 Introduction

Semiconductor nanocrystals (NCs) are technologically relevant materials due to their size-tunable optoelectronic properties.^1,2^ Historically, lead, cadmium, and mercury-based binary systems have been at the research forefront.^3^ However, their high environmental toxicity has led to restrictions by the European Union.^4^ Therefore, there is a need for less-toxic NC alternatives. In this regard, the list of "nonrestricted" materials for optoelectronic devices is limited. For instance, InP is the principal alternative to replace Cd-containing NCs.^5^ However, the scarcity of indium and its environmental concerns highlight the need for other options.^6^ Similar concerns apply to many emissive NCs operating in the visible and near-IR region, leaving an opportunity for more sustainable solutions. A viable strategy to find Pb/Cd-free alternatives that perform as well as their restricted counterparts is to increase the complexity of NCs by exploring multinary compositions, which would drastically increase the number of candidate materials compared to binary materials alone.^6^

In this regard, Ag-containing NC alternatives have garnered attention in the past years due to their low elemental toxicity and large variety of phases.^7–9^ However, only a limited number of Ag-based systems has been explored through colloidal chemistry and further advancements have been hampered by challenges including limited thermal conductivity, poor phase stability, and low photoluminescence quantum yields.^10,11^ An attractive family in the compositional space of Ag-based ternary materials is the argyrodites family, which share a common formula of *A*_(12–*n*)*/m*_^m+^ *B* ^*n*+^X_6_^2–^, (where A = Li, Ag, Cu; B = Si, Ge, Sn; and X = S, Se, Te with *m* and *n* as valence states of *A* and *B*, respectively).^12^ Bulk argyrodites have complex and flexible lattices: cation disorder, for instance, is a major feature of these lattices because of their weak metal-to-chalcogen bonding.^12–15^ Still, controlling cation disorder and ionic conductivity renders materials useful in band gap tunability,^16^ photovoltaics and battery materials,^8,17,18^ and thermoelectrics,^19^ making argyrodites highly appealing materials.

Among the naturally occurring argyrodites, canfieldite (Ag_8_SnS_6_) is a highly attractive optoelectronic material due to its direct and narrow band gap (1.1–1.4 eV) and high absorption coefficients (10^4^–10^5^ cm^–1^).^20–23^ Indeed, some preliminary synthetic routes exist to obtain Ag_8_SnS_6_ as colloidal NCs and inks (ranging from 7 to 13 nm),^22,24^ one in which the modification of optical properties via quantum confinement has been suggested.^23^ However, a significant challenge concerns the identification of argyrodite phases at the nanoscale (<10 nm). This stems from the tendency of Ag–Sn–S and related systems to produce multiple phases with similar compositions and polymorphs which, combined with broadened X-ray diffraction (XRD) patterns due to the finite size of NCs, complicates structure identification.^23,25,26^ In particular, bulk Ag_8_SnS_6_ is known to crystallize into two polymorphs, the room-temperature orthorhombic (*Pna*2_1_) and the high-temperature cubic (*F*4̅3*m*) one above ∼170 °C. This phase transition is attributed to the onset of superionic conductivity, which arises due to the thermal disordering of the cationic positions of Ag^+^ in the crystal.^14,21,27,28^

Polymorph identification and functionality are critical considerations in the development of multinary NCs.^13,29–31^ For instance, the presence of polymorphs in systems such as Cu_2_ZnSnS_4_ and AgBiS_2_ can profoundly impact device performance.^32–34^ Additionally, a significant challenge has been found in the phase identification of Ag–Sn–S systems with added zinc (e.g., Ag_8_SnS_6_ vs Ag_2_ZnSnS_4_). This difficulty arises from the ambiguous experimental stoichiometries and diffractograms observed,^7,35–37^ complicating structural characterization and posing the question as to whether Zn^2+^ is incorporated into Ag_8_SnS_6_ particles to yield Ag_2_SnZnS_4_ in any measurable quantity. Considering the polymorphic nature of argyrodites and the expected roles of cationic mobility and structural distortion,^3,22^ clarification of these points is of central importance to establish design principles in this family of low elemental toxicity NCs with useful optoelectronic properties.

Here, we investigate the growth and controversial structure of NCs in the extended argyrodite family that contains Ag, Sn, Zn, and S as an attractive, Pb/Cd-free material system. We synthesized sub 7 nm Ag_8_SnS_6_ NCs, with emission in the λ: 750–830 nm region. Under our reaction conditions, we can also observe the formation of an emissive, cluster-like species (∼1.5 nm in diameter) with peak emission at λ: 630 nm, which is the first observation of such a species to the best of our knowledge. Then, we resolve the phase identification challenge in Ag–Sn–S systems by employing elemental analysis techniques as well as high-resolution (scanning) transmission electron microscopy (HR-STEM) and synchrotron X-ray total scattering for pair distribution function analysis (PDF). Our findings demonstrate that distinguishing between the orthorhombic and the cubic polymorphs of canfieldite becomes unimportant at the nanoscale because NCs adopt a canfieldite-like phase with a pseudo-orthorhombic structure of Ag_8_SnS_6_. Finally, we exploited the same set of techniques to retroactively investigate canfieldite NCs prepared in the presence of Zn^2+^, which were previously believed to be a different material (i.e., pirquitasite Ag_2_ZnSnS_4_). Contrary to expectations, we demonstrate that the incorporation of Zn^2+^ does not change the canfieldite-like phase but it contributes to boosting the emission. Overall, this investigation into the synthesis, optical properties, and structural composition of Ag–Sn–S NCs expands our understanding of superionic semiconductor materials at the nanoscale, opening up new possibilities for material design within the argyrodite family.

## 2 Results and Discussion

### 2.1 Optical and Structural Properties of NCs in the Ag–Sn–S (ATS) Material Family

#### 2.1.1 Synthesis and Optical Properties of ATS Products

We first focused on the synthesis and characterization of the optical properties of ATS NCs with the goal of learning about this underexplored system as well as having optical information that could be correlated to our structural findings. To synthesize ATS NCs, we modified our reported procedure to target pure ATS NCs (see SI Section 1).^35^ Our ATS syntheses yield products with sizes 2.1–6.9 nm under low-resolution TEM (Figure S1). The size can be tuned by varying the growth time and reaction temperature while the isolation requires a size-selective precipitation (see SI Section 1 and Figure S2). Visually, solutions with smaller ATS NCs have a bright red color while the fraction of larger NCs has a dark brown tint, while optical spectroscopy reveals corresponding shifts in their absorption spectra emission peak (Figure 1a). Notably, the peak emission at of the smaller species is located with remarkable consistency at λ: 630 nm across syntheses (Figures 1a and S3). By comparison, the emission peaks from larger ATS NCs are in the λ: 700–750 nm range (Figure 1 a), consistent with a size effect, but vary between batches and fractions (Figure S3). Further, samples of the smaller red-looking species are significantly unstable and convert to the brown species with emission near λ: 740 nm (typical of larger ATS NCs) over a ∼12 h period (Figure S4). These observations align with the behavior of thermodynamically unstable cluster species seen in other material systems, suggesting that such species might be present in our synthesis.^38–40^

**Figure 1:**
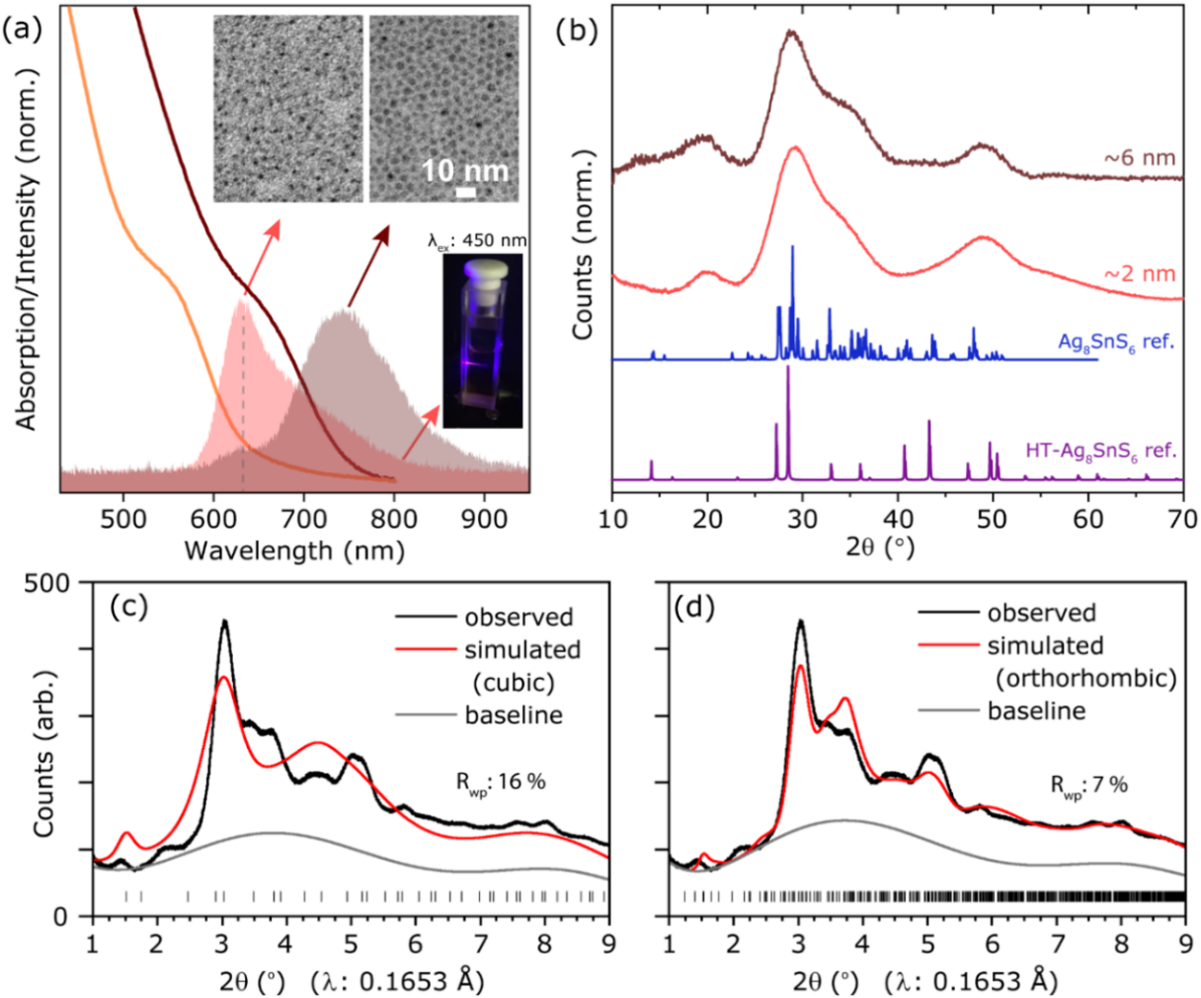
Optical characterization, TEM images, and diffractograms for ATS NCs. (a) Absorbance and photoluminescence spectra for ATS NCs purified through a size-selective precipitation. The cluster-like species have a peak emission at 630 nm. The inset shows bright-field TEM images (120 kV) of these two NCs samples. The bottom inset shows a cuvette containing cluster-like ATS NCs being illuminated by λ: 450 nm light. (b) Experimental PXRD diffractograms for ATS NC samples of sizes 2 and 6 nm, acquired for 24 and 6 h, respectively. The reference patterns for orthorhombic (PDF 00–038–0434) and high-temperature cubic (PDF 04–002–4840) canfieldite are shown in blue and purple. Rietveld fit for patterns acquired on 6 nm NCs for the cubic (c) and orthorhombic (d) phase using identical baseline functions. *R*_wp_ represents the weighted profile residual.

Elemental analysis through STEM-EDX and XPS of ATS NCs with an average size of 6.0 nm show a stoichiometry of 4.0:1.0:3.2 and 3.3:1.0:3.6 (Ag:Sn:S), respectively (Figures S5, S6, and Table S1). These results are *not* consistent with the theoretical stoichiometry of bulk canfieldite Ag_8_SnS_6_ and could align with other phases in the Ag–Sn–S system such as monoclinic Ag_2_SnS_3_ or Ag_4_Sn_3_S_8_ (Figure S7c). However, major variations in the theoretical stoichiometry can be observed in NCs due to faceting, where the atoms missing from the truncated lattice can be strongly biased by facet-specific surface energies and ligand interactions.^41–43^ Thus, an orthorhombic canfieldite structure for our ATS NC is similarly plausible and the structure cannot be attributed solely through compositional analysis.

Powder X-ray Diffraction (PXRD) measurements on ∼2 and ∼6 nm NCs are ambiguous and do not allow us to confidently assign a phase, showing significantly broadened Bragg reflections (Figure 1 b). Indeed, the experimental patterns look unusually similar considering that one would expect a 3-fold sharpening in the signal as per the Scherrer equation, assuming single-domain NCs (Figure S7a, vide infra).^44^ We then acquired high-resolution PXRD data to test the cubic and orthorhombic models, and performed Rietveld refinement on the resulting diffractograms (see SI Section 2 for details). Our analysis shows that the cubic structure cannot index the second feature just above 2° and fits poorly to the rest of the reflections (Figure 1c). On the other hand, the orthorhombic structure better describes the features of the diffraction pattern but also does not give a good match (Figure 1d). Overall, due to extreme broadening of Bragg reflections and limited description of the diffraction features by either model, neither can accurately describe the structure of the particles, and further analysis of the local structure is required, vide infra.

#### 2.1.2 Single-NC Analysis via HR-TEM

In order to simplify our structural analysis and facilitate comparison to literature data, we decided to focus on larger (>5 nm, Figure 2a) ATS NCs, which are grown at 95 °C (see Methods). Since structural analysis using PXRD yields ambiguous results regarding the phase, we acquired single-NC images by means of HR-TEM to probe the crystalline structure of ATS NCs (Figure 1b). In the TEM images we note ATS NCs that display signs of polycrystallinity (Figures 2b and S8). We identify NC structures that appear to be composed of smaller triangular crystalline domains of height ∼2.1 nm (Figure 2c). Such triangular domains are in the length order of 2–3 canfieldite unit cells and aggregate in an ordered fashion, resembling the behavior of some gold nanoclusters, which undergo aggregative growth to form star-shaped particles.^45^ Furthermore, we noticed that these polycrystalline NCs coalesce under electron beam irradiation into single, but faulted, crystalline domains (Figure S8), aided by the high intrinsic mobility of Ag atoms in argyrodite materials.^12,13,15^ These results provide insight into the formation mechanism of ATS NCs in which a stepwise process concerning the preformation of cluster-like species followed by aggregative growth and coalescence could be at play. In fact, this hypothesis can explain the optical behavior that we see in both the absorption and PL spectra in Figure 1a where the emission of cluster-like species at 630 nm and the excitonic features broaden as NCs grow. Additionally, polycrystallinity and faulting in NCs have been correlated with electronic trap states, quenching the PL.^46,47^ This also aligns with the low PLQY value (<1%) that we measure for these particles. The bottom scheme in Figure 2 summarizes our proposed formation mechanism and aggregative growth step for ATS NCs. Structurally, such degree of polycrystallinity and stacking faults are known to affect NC phase identification, provoking significant broadening and changes to PXRD diffractograms.^48–50^ Thus, our observations provide a partial explanation of the breadth and unexpected size-independent of the Bragg reflections in our PXRD measurements (Figures 1b and S7a). However, bypassing polycrystallinity likely requires reaction temperatures exceeding 400 °C as suggested on our temperature-dependent PXRD measurements (Figure S9). While this falls outside the scope of our current study, optimizing crystallinity under milder conditions presents an important avenue for future research.

**Figure 2:**
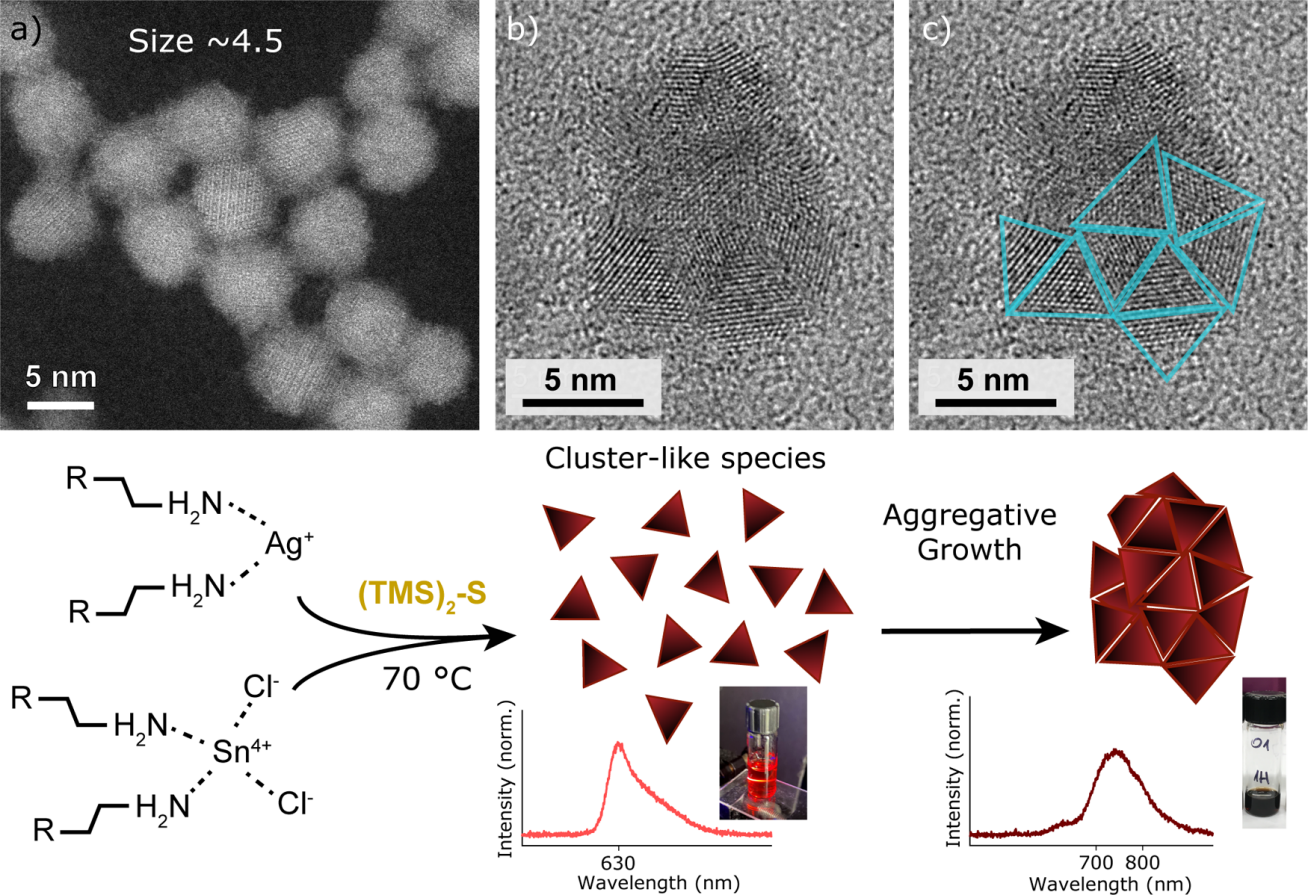
TEM images and reaction scheme for ATS NCs. (a) HR HAADF STEM image showing ATS NCs of size ∼4.5 nm. (b, c) Bright-field HR-TEM image illustrating a polycrystalline ATS NC composed of cluster-like species. The cartoon at the bottom depicts the proposed formation mechanism of cluster-like species and their aggregative growth leading to larger, polycrystalline ATS NCs.

We then performed FFT analysis to probe the phase of ATS NCs. Through our measurements, we identify images displaying the [211] and the [110] zone axes of canfieldite with *d*-spacing 0.635, 0.388, and 0.558 nm (Figure 3a–e). These *d*-spacing values have an excellent correlation with reported values for the high-temperature cubic phase of canfieldite where silver is highly disordered.^14^ Additionally, we identify *d*-spacings 0.311 and 0.301 nm that correlate with main reflections (022) and (411) of the orthorhombic phase of canfieldite (Figure S10).^51^ However, despite the distinction in space groups, both phases share a common Sn framework (Figure 3e) with the primary difference being the degree of disorder in the silver atoms. As a result, these measurements robustly confirm that the position of heavy Sn atoms in these NCs is compatible with canfieldite. These findings also corroborate that the coalescence of polycrystalline NCs into single crystalline domains does not change the material composition. However, the weaker and broader reflections anticipated from the disordered Ag atoms mean that unraveling the structure of ATS NCs remains challenging via HR-TEM measurements alone. Therefore, we pursued further structural studies using pair distribution functional analysis because this technique is sensitive to the local coordination environment of atoms in the lattice.

**Figure 3:**
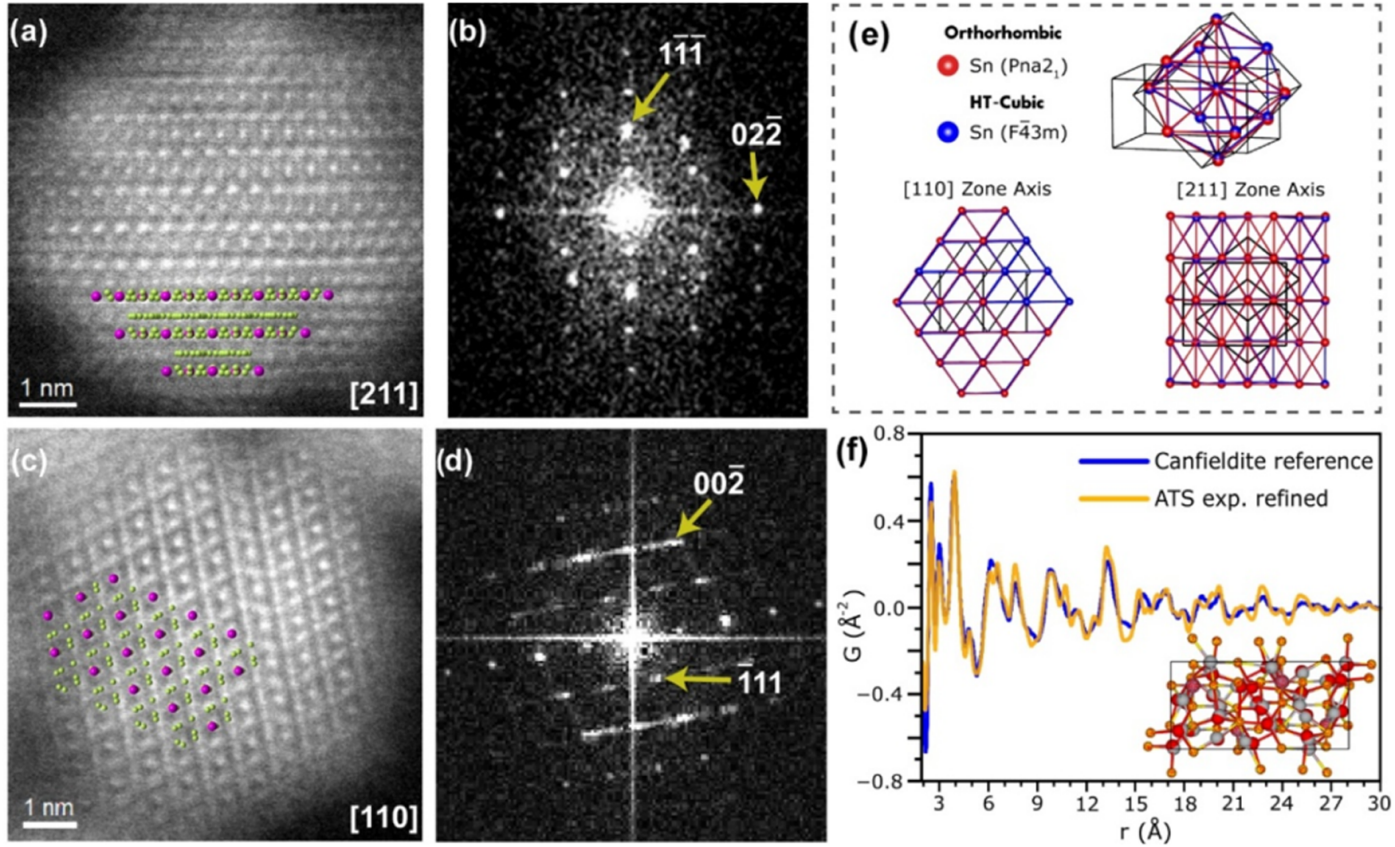
HR STEM images, corresponding FFT patterns, and PDF scattering pattern for ATS NCs. (a) HR-HAADF-STEM image displaying the [211] zone axis of a canfieldite NC with the atomic map overlaid on the image for better visualization of the lattice with Sn in pink and Ag in green, (b) associated FFT pattern. (c) HR-HAADF-STEM image displaying the [110] zone axis for a canfieldite NC and (d) associated FFT pattern. (e) Atomic structural models showing the framework of Sn atoms in orthorhombic (red, PDF 00–038–0434) and cubic (blue, PDF 04–002–4840) canfieldite overlaid. Sn–Sn bonds do not exist in the original lattice but were simulated for better visualization of the indistinguishable frameworks. (f) PDF analysis of ATS NCs. The Canfieldite reference model is fitted to the experimental data with refined Ag positions. The inset shows the structural model with refined Ag positions in red compared to the original atoms in gray.

#### 2.1.3 Structural Analysis of ATS NCs via Pair Distribution Function

We performed synchrotron X-ray total scattering measurements with PDF analysis on ∼6 nm ATS NCs to get further information about the structure of our NCs. The experimental PDF displays a loss in spatial coherence at very low distances (∼30 Å), as seen by the dampening of the peak intensity (Figure 3f). This evidence suggests that the ordered domain size of our ∼6 nm ATS NCs is of 3 nm, correlating with our observations of polycrystalline NCs (Figure 2b,c) and providing further support for our proposed growth model via aggregative growth (scheme in Figure 2). To investigate the structure of our particles, the PDF data were initially fitted using the model of bulk orthorhombic canfieldite (Figure 3f). Our model was refined by adjusting the position of all Ag atoms while keeping both the unit cell parameters as well as the position of Sn and S atoms identical to the original orthorhombic model. This refinement described the local structure (2–10 Å) of ATS NCs with a goodness-of-fit (*R*_w_) of 0.257, representing the main features of the experimental PDF without major differences from the original canfieldite model. This suggests that the local coordination environment of our ATS NCs is similar to canfieldite. However, considering the similarity in the Sn position between the orthorhombic and cubic structures (Figure 3e), and the highly disordered position of Ag atoms in the high-temperature cubic model, a more intricate modeling strategy was implemented to try to better capture and describe the local structure of ATS NCs. First, both orthorhombic and cubic models were evaluated in terms of their capacity to describe the intermediate-to-long-range structure (10–30 Å), which is subject to long-range averaging of different local environments in a statistical structure.^52^ In this analysis, both models described the overall features similarly (Figure S11): cubic model with disordered Ag sites (*R*_wp_ = 28%), orthorhombic model with discrete Ag sites (*R*_wp_ = 29%). Thus, it is possible that the description of the local structure could be better explained by a model that contains disordered Ag atom sites. To evaluate this hypothesis, we constructed four different models comprised of the following basis features: SnS_4_ tetrahedra modeled as rigid bodies. The Sn atom was fixed while the vertices of the tetrahedra (S atoms) were allowed to rotate around the center of mass, and Ag atoms with positions refined freely except for an "antibump" constraint (more details about model characteristics are found in SI Section 4 and Table S2). The four models are referred to as pseudocubic, orthorhombic, pseudo-orthorhombic I, and pseudo-orthorhombic II. In our analysis, we observed that the system was insensitive to the rotation of SnS_4_ tetrahedra due to their low contribution to the PDF signal. Thus, this feature was set to fixed positions in subsequent calculations, a reasonable assumption, considering that the framework of Sn atoms is identical in both polymorphs (Figure 3e). Furthermore, to account for Ag atom disorder, our models required discrete Ag positions.

Considering these two requirements, the pseudocubic (Figure 4a) and orthorhombic (Figure 4b) models underperform in the fit compared to the pseudo-orthorhombic models (Figure 4c,d). This suggests that Ag prefers an arrangement of SnS_4_ tetrahedra that are slightly distorted away from their relative positions compared to the cubic model, making it difficult to clearly distinguish the structural tiling. Such distortion is feasible if we consider that the distribution of local environments is more diverse than what can be captured in this simple small-box model. The pseudo-orthorhombic II (P1) structure was used for Rietveld analysis of the ATS diffraction pattern (Figure S12) showing the capabilities of the model to describe the broad reflection features of the sample, as described by preliminary Rietveld analysis shown above.

**Figure 4:**
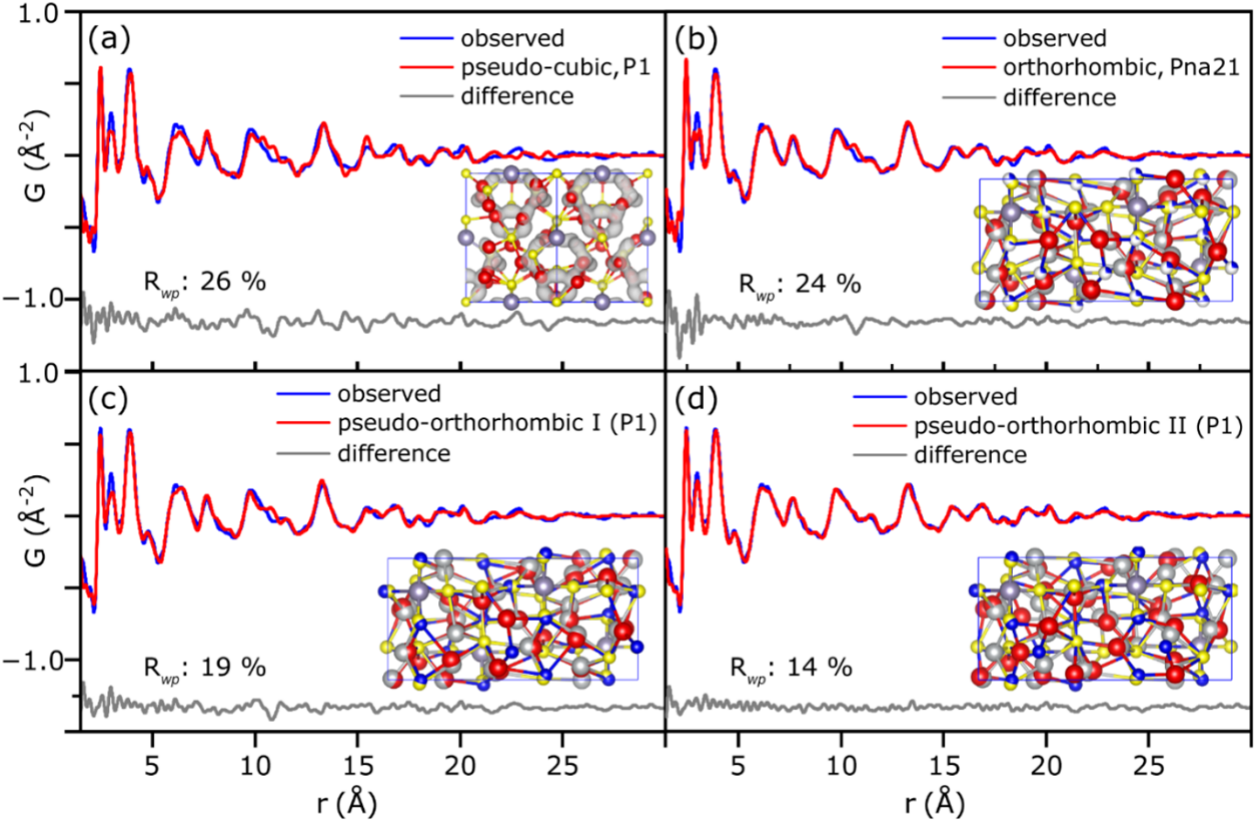
Results of real-space fits and models of the modified cubic and orthorhombic structures to the local and intermediate range structuring as observed in the PDF for (a) pseudocubic, (b) orthorhombic, (c) pseudo-orthorhombic I, and (d) pseudo-orthorhombic II. Each inset structure is an overlap of the original position of Ag (gray), Sn (purple), and S (yellow) atoms and the refined positions (red). *R*_wp_ represents the weighted profile residual. In panel (a), the isosurface describes the disorder of Ag atoms in the original cubic model.

Taken together, our models represent a description and compatibility of atomic positions with the local structure of ATS NCs, which in reality is a much broader distribution of local structures. Ultimately, the PDF analysis suggests that (1) the local and intermediate-range structures of ATS NCs are compatible with a canfieldite-like structure, (2) the true structure likely comprises site disorder of the Ag atoms, distributed along a tiling of SnS_4_ tetrahedra and S atoms, and (3) SnS_4_ tetrahedra can rotate on site in the structure, however its PDF signal does not appear sensitive enough to finely resolve the nature of their orientations.

#### 2.1.4 Structural Analysis of Cluster-like Species

We performed total X-ray scattering measurements and PDF analysis on ATS cluster-like species to corroborate the structure and get additional insight into their size. Given that these species would degrade rapidly (Figure S4), we sought a "postsynthetic" stabilization procedure using ZnBr_2_ to extend their bench lifetime despite their minimal compositional effects, which do not affect the structural symmetry (see Section 2.2). The PDF data matched that of the two pseudo-orthorhombic models at short distances (Figure S13). However, a major difference between large ATS NCs and these cluster-like samples is the rapid and sharp signal dampening at ∼5 Å and loss of spatial coherence at ∼15 Å. This value is on the order of a few canfieldite unit cells (*a*: 15.3, *b*: 7.5, *c*: 10.7 Å). Hence, this result supports our hypothesized formation of a small, cluster-like species with canfieldite structure. Additionally, Figure S12c shows that the comparison between the experimental diffraction pattern of cluster-like species and that of the pseudo-orthorhombic (P1) structure model after refinement through PDF analysis. Their resemblance demonstrates the ability of our model to describe the structure of cluster-like species. Overall, the PDF analysis of our ATS particles reveals clear structural deviations compared to bulk canfieldite, suggesting that Ag and Sn atoms play an important role in the optical properties that emerge at the nanoscale (i.e., red emission). Furthermore, our PDF analysis displays evidence for the presence of canfieldite cluster-like species, which expands our understanding of the formation mechanism of larger NCs in this material family.

### 2.2 Effect of Zn on the Structural and Optical Properties of ATS NCs: Solving the Phase Identification Controversy

After unraveling the structural characteristics of pure ATS NCs, we proceeded to tackle the structural controversy into the phase identification of Ag–Sn–S systems with added Zn^2+^ (which we will refer to as ATS@Zn). More specifically, our motivation stems from the fact that our groups and others^7,35,37^ have encountered phase identification challenges when studying the synthesis and physicochemical properties of supposed pirquitasite (Ag_2_ZnSnS_4_) NCs. Similar to the discussion of canfieldite NCs above, the challenges arose in part due to the severe broadening of Bragg reflections.^7,35,53,54^

#### 2.2.1 Synthesis and Optical Properties of ATS@Zn Products

We introduced Zn^2+^ into ATS reactions (see Methods), following the methods previously reported to yield pirquitasite NCs. ATS@Zn samples were prepared using our reported procedure^35^ as well as a previously developed synthesis^7^ (see Methods). We will refer to these NCs as ATS@Zn-1 and ATS@Zn-2, respectively. These samples were of similar average size (∼5.0 nm, under low-res TEM) with comparable stoichiometry to ATS NCs and had a photoluminescence maximum at ∼790 nm (Figure S14 and Table S1). Visually, we can see that the presence of Zn^2+^ grants a more-gradual NC growth in ATS@Zn-1 products. We speculate that Zn^2+^ regulates the kinetics by acting as a Z-type ligand on surface sulfur, as seen in other materials.^55,56^ Zn^2+^ may also slow growth by partially exchanging with Ag^+^, reducing free Ag^+^ availability for lattice incorporation. Further, the excitonic absorption feature is more defined in ATS@Zn-1 (Figure 5a) relative to ATS NCs (Figure 1a). This allows us to better follow the growth (inferred from a progressive bathochromic shift) of these species (Figure S15). The peak of the excitonic absorption in the earliest aliquots is at 2.36 eV (Figure 5a), representing an optical gap that is 1.11 eV larger than the band gap of bulk canfieldite. We also observe a PLQY enhancement from ∼1 to 5% when ATS NCs are treated with ZnBr_2_ "postsynthetically", matching previous reports and suggesting that in our case, Zn may also passivates surface traps.^57–59^ In addition, elemental analysis using XPS shows average elemental ratios of 2.2:1.0:0.7:3.9 for ATS@Zn-1 NCs (Ag:Sn:Zn:S, Figure S5 and Table S1). This observation is consistent with previous comparable studies,^7,35,37^ wherein it was used to support claims of the synthesis of pirquitasite (Ag_2_SnZnS_4_) as the primary phase. This is because the roughly 1:1 ratio between Sn and Zn is consistent with the bulk stoichiometry of pirquitasite. However, considering that the unit cell of canfieldite contains only four Sn atoms, and that larger NCs are composed of aggregates of smaller domains (vide supra), we speculate that even surface-bound Zn could contribute significantly to the stoichiometry of the overall NC. Any de facto replacement of Ag by Zn could also cause stoichiometric deviations. Therefore, we explored whether a canfieldite core passivated with Zn on the surface could also explain the composition that we observe.

**Figure 5:**
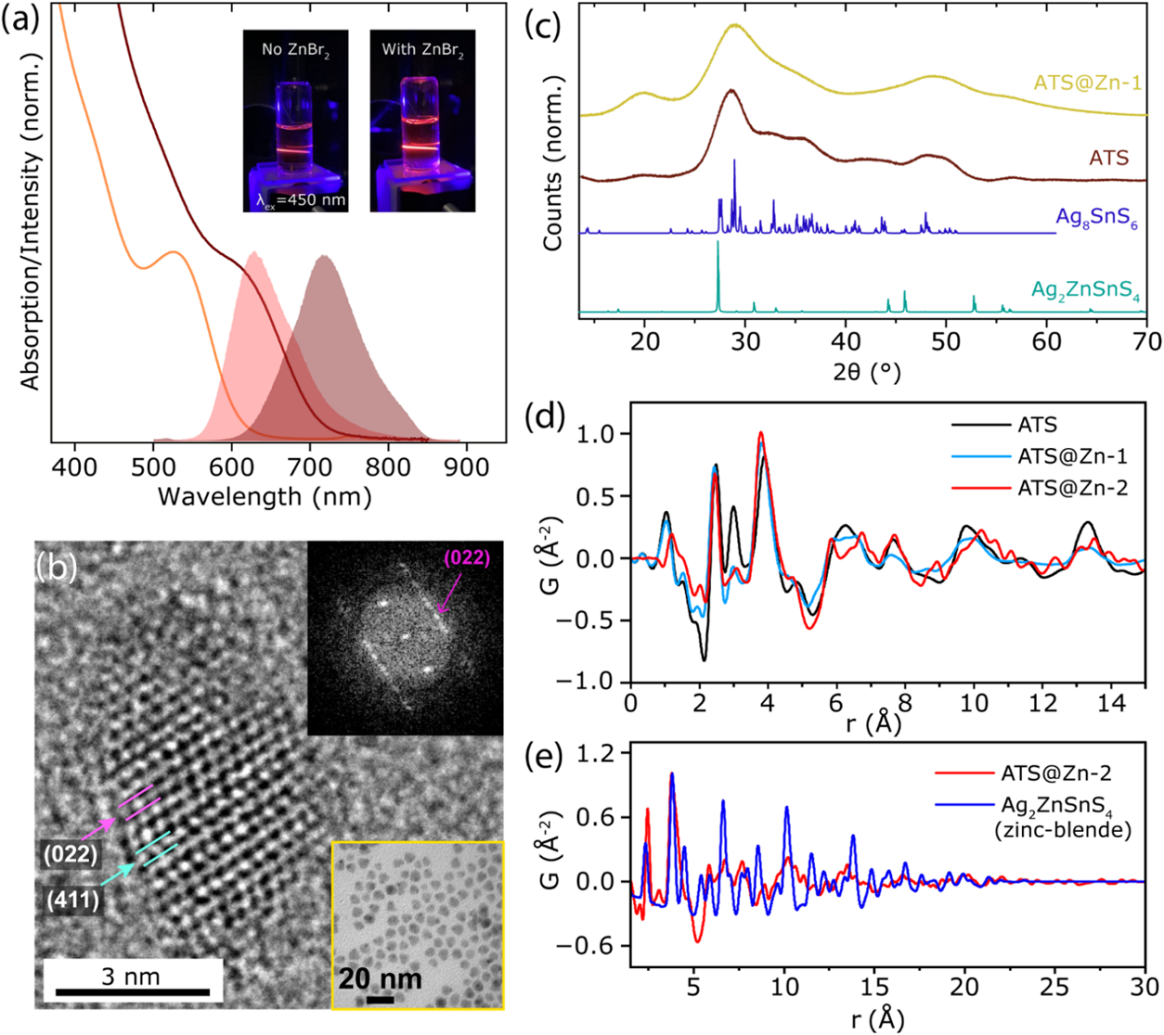
Optical and physical characterization for ATS NCs containing Zn. (a) Optical characterization for two ATS@Zn-1 NC sizes. The absorbance spectrum of the smallest NCs (orange trace) shows a well-defined excitonic feature and an emission peak at ∼630 nm seen in the red trace and the inset. The brown traces display the absorbance and photoluminescence spectra for larger ATS@Zn-1 NCs. (b) HR-TEM image and corresponding FFT pattern for a single ATS@Zn-1 NCs, displaying *d*-spacing values that correlate with canfieldite. The inset shows a low-resolution TEM image of ATS@Zn-1 NCs of size ∼6 nm used for PDF analysis. (c) HR-PXRD data for ATS and ATS@Zn-1. (d) PDF scattering plots for ATS, ATS@Zn-1, and ATS@Zn-2. (e) PDF scattering plot comparing ATS@Zn-2 to bulk Ag_2_ZnSnS_4_. The local structure of ATS@Zn-2 NCs is not compatible with the zinc-blende phase.

#### 2.2.2 Single-NC Analysis via HR-TEM

We conducted HR-TEM on ATS@Zn-1 NCs to obtain structural information about the impact of Zn passivation and/or incorporation. The images show similar results to ATS NCs in terms of *d*-spacing values where we identify the (411) and (022) planes of canfieldite (Figure 5b). Additionally, we attain further insight into the preferred planes for the formation of stacking faults (see Figure S16 and associated discussion). However, it is important to highlight that relying solely on *d*-spacing values can lead to ambiguity over the phase distinction between pirquitasite and canfieldite. This is because the (112) and (022) lattice planes (and main PXRD reflections) in pirquitasite and canfieldite respectively have the same interplanar distance (i.e., 0.31 nm). Consequently, based on our HR-TEM measurements we cannot confirm the presence of pirquitasite or the inclusion of Zn^2+^ in the lattice in ATS@Zn-1 products. Instead, our results have a better correlation with the structural characteristics of a canfieldite-like phase.

#### 2.2.3 Crystallographic Analysis for ATS@Zn NCs via Pair Distribution Function

We used synchrotron total X-ray scattering measurements on ATS@Zn to investigate how Zn^2+^ affected the local structure of ATS and assess whether the formation of pirquitasite was feasible under our reaction conditions, as well as under previously reported conditions. Although the information extracted through high-resolution PXRD is limited due to the breath of the Bragg reflections, comparing the diffraction pattern of ATS to that of ATS@Zn-1 NCs provides qualitative insight. Specifically, all the reflections seen for ATS@Zn-1 NCs are notably broader than those of ATS NCs (Figure 5c), which would arise if the addition of Zn resulted in smaller effective crystal domains. PDF analysis of ATS@Zn-1 NCs reveals a signal that resembles that of pseudo-orthorhombic canfieldite-like models (Figures 5d and S16). However, the nearest-neighbor distances for Sn–S and Ag–S pairs in ATS@Zn-1 (∼2.421 Å) are shorter than those in pure ATS (∼2.464 Å). Given that Zn^2+^ has a smaller ionic radius than Ag^+^, this contraction is consistent with the formation of Zn–S pairs. Additionally, the peak at ∼3 Å, primarily attributed to Ag–Ag pairs in ATS, exhibits significantly reduced intensity. We attribute this weakening to the introduction of Zn–Ag pairs because Zn has a lower scattering power, diminishing the overall signal. Similarly, the peak at ∼4 Å, dominated by Ag–Sn pairs, shifts to a shorter distance. We associate this with the incorporation of Zn–Sn pairs in ATS@Zn-1, leading to a reduced next-neighbor distance in Zn-(S)-Sn and Ag-(S)-Sn configurations. Furthermore, the higher radial distances in ATS@Zn-1 appear slightly broader, and the domain size is smaller (∼2.5 nm) compared to ATS (Figures 5d and S17), indicating greater structural disorder. This disorder likely stems from a wider distribution of local environments induced by Zn^2+^ incorporation throughout the whole ATS structure. Synthetically, this means that the inclusion of Zn^2+^ through Ag^+^ atom displacement must occur *after* the formation of the ATS core, given that Zn^2+^ is introduced "postnucleation", rendering our NCs a highly appealing platform for cation exchange.

We then proceeded to analyze NCs from a second reported procedure to achieve pirquitasite^7^ (labeled ATS@Zn-2 here). The NCs used were of similar size and emission to our ATS@Zn-1 samples (Figure S14c,d). Unexpectedly, the PDF of ATS@Zn-2 NCs coincide with a canfieldite-like phase and **not** zinc-blende pirquitasite (Figure 5d,e). In fact, the zinc-blende fit cannot even remotely describe neither the local structure nor larger distances in ATS@Zn-2 samples (Figure 5e), evidencing that this structure is highly inaccurate to characterize these NCs. Conversely, comparing the experimental data for ATS@Zn-2 to the canfieldite-like model shows that the local structure up to 5 Å is highly similar to ATS and ATS@Zn-1 with small Zn-related modifications. We also noticed slight deviations beyond the local structure (>5 Å) in ATS@Zn-2 compared to ATS@Zn-1 (Figure S17). We attribute this small difference to the way in which Zn^2+^ is included in the structure. In ATS@Zn-1 the Zn^2+^ inserts into the lattice after the ATS core is formed at 70 °C, while in ATS@Zn-2 the Zn^2+^ is present in the reaction before the sulfur injection at 160 °C. Overall, the main features of the PDF of ATS@Zn-2 are highly compatible with a canfieldite-like model. Hence, our results indicate that the formation of true pirquitasite NCs might have a higher thermodynamic barrier relative to canfieldite and, thereby, be more difficult to access with conventional synthetic routes.

## 3 Conclusions

We investigated the synthesis and structural characterization of canfieldite (Ag_8_SnS_6_, ATS), an underexplored, superionic material system, in its nanoscale form. First, we found emissive cluster-like ATS species that are involved in the formation and growth of larger NCs via aggregative growth and coalescence. The red emission is not observed in bulk canfieldite and is likely related to size reduction. These findings set a baseline for the design of procedures for the synthesis of superionic argyrodite NCs. We performed in-depth structural studies via HR-STEM to unravel the structural features of ATS NCs. We find that the breadth in reported PXRD diffractograms likely arises due to polycrystallinity. Additionally, we find that the structure of our NCs is compatible with a canfieldite lattice, and we show that the framework of heavy atoms (i.e., Sn) is indistinguishable between canfieldite polymorphs, an overlooked fact that frustrated previous interpretations of the phase of Ag–Sn–S materials. We modeled canfieldite polymorphs through PDF analysis and found that the structure of our NCs cannot be described through a single bulk-derived model but instead requires an averaged canfieldite-like phase that accounts for structural modifications. Such structural distortions observed in nanocrystalline samples of ATS correlate with the onset of photon emission, highlighting a possible role of nanoscale structural perturbations in dictating optoelectronic properties. Our results emphasize the importance of probing the local structure of NCs to fully understand and tailor the functionality of nanomaterials. Future work incorporating molecular dynamics simulations could provide deeper mechanistic insights into the dynamic behavior and structural flexibility of these systems.

Finally, we solved a standing controversy regarding the phase identification in canfieldite-pirquitasite (Ag_2_ZnSnS_4_) Ag–Sn–S@Zn systems using a combination of HR-TEM and pair distribution function. Critically, we identify that HR- measurements are unsuitable to confirm the presence of pirquitasite because of the similarity in *d*-spacing value with canfieldite. We performed total scattering measurements with PDF analysis on various Ag–Sn–S-Zn samples and confirm the formation of canfieldite as the main phase in all synthesized products. We find that the addition of Zn^2+^ into the canfieldite syntheses does not grant the formation of pirquitasite Ag_2_ZnSnS_4_, as previously thought, even when following reported higher-temperature procedures. Instead, we find that Zn^2+^ replaces Ag^+^ throughout the lattice, making our particles an appealing platform for cation exchange procedures.

Taken together, our study elucidates the design and exploration of argyrodite nanostructures, revealing critical synthetic and structural insights that advance the understanding of their unusual nanoscale behavior. Future work should employ ab initio molecular dynamics to resolve static versus dynamic disorder, refining knowledge of structural distortions governing optoelectronic properties. Additionally, NMR studies will help reveal surface faceting and key binding sites to enhance the functionality of this material system. These findings will inspire synthetic and computational efforts to uncover emerging properties in underexplored multinary systems, moving beyond binary materials, while discovering effective dopants and surface stabilization techniques will be key to enhancing their performance.

## Supplementary Material







## Notes

ATS synthesis tutorial (Video 1) (MOV)
ATS-Zn-1 synthesis tutorial (Video 2) (MOV)
Synthetic methods and characterization techniques. Also, there are two tutorial videos demonstrating the synthetic procedures for ATS and ATS@Zn-1 NCs (Section 1); physical and optical characterization images for our products as well as a detailed description of the Rietveld refinement (Section 2); TEM and diffraction data (Section 3). PDF analysis including the parameters used for the modeling of the structure refinements. The CIF files for our four refined structures are also attached (Section 4); physical and optical characterization for products synthesized with added zinc (Section 5) (PDF)
