# Supplementary material for: Synthesis and Structural Analysis of an Emissive Colloidal Argyrodite Nanocrystal: Canfieldite Ag8SnS6
Source: J Am Chem Soc. 2025 Jul 30;147(32):29413–22. doi: 10.1021/jacs.5c09495 (PMC12356587; doi:10.1021/jacs.5c09495)
Supplement: Supplementary file 3 [file ja5c09495_si_003.pdf]

# Supporting Information

# Synthesis and Structural Analysis of an Emissive Colloidal Argyrodite Nanocrystal: Canfieldite $Ag_8SnS_6$

Francisco Yarur Villanueva,[1,2] Victor Quezada Novoa,[3] Pascal Rusch,[1] Stefano Toso,[1] Maxwell W. Terban,[4] Yurii P. Ivanov,[1] Joaquin Carlos Chu,[2] Maxine J. Kirshenbaum,[2] Ehsan Nikbin,[5 1] Maria J. Gendron Romero,[2] Mirko Prato,[1] Giorgio Divitini,[1] Jane Y. Howe,[5] Mark W.B. Wilson,[2*] Liberato Manna[1*]

[1]Istituto Italiano di Tecnologia, Via Morego 30, 16163, Genova, Italy

[2]Department of Chemistry, University of Toronto, Toronto, Ontario, M5S 3H6, Canada

[3]Department of Chemistry and Biochemistry and Centre for NanoScience Research, Concordia University, 7141 Sherbrooke Street West, Montréal, Quebec H4B 1R6, Canada

[4]Momentum Transfer GmbH, Luruper Hauptstraße 1, 22547 Hamburg, Germany

[5]University of Toronto, Department of Materials Science and Engineering, Toronto, Ontario, M5S3E4, Canada

Contents

**Section 1.** Methods ........ 2

**Section 2**. Physical and Optical Characterization for ATS NCs ........ 6

**Section 3**. Supporting PXRD, TEM, and electron diffraction data ........ 11

**Section 4**. PDF analysis. ........ 14

**Section 5**. Physical and optical characterization for ATS@Zn NCs ........ 16

**Section 1.** Methods

*Chemicals:* Silver (I) Chloride [AgCl], Tin (IV) Chloride [$SnCl_4$], Zinc (II) Bromide [$ZnBr_2$], hexamethyldisilathiane [$(TMS)_2$-S], Oleylamine [OLA 98%], Toluene 99%, Ethanol [EtOH, 99.9%], Methanol (99%), Hexane [≥99%], dimethylformamide [DMF 98%], and tetrabutylammonium bromide (TBABr, 98%) were purchased from Sigma Aldrich. All chemicals were used as received without further purification except for OLA, which was degassed at 125 °C under vacuum for 3 hours and stored under argon.

*Synthesis of ATS and ATS@Zn-1 Nanocrystals (adapted from Yarur Villanueva et al.)[1]:* A 25 mL 3-neck round-bottom flask was loaded with AgCl (42 mg, 0.30 mmol), sealed with a rubber septum and connected to a Schlenk line *via* condenser. The flask was evacuated, and 8 mL of degassed OLA 98% were added while stirring. This flask is placed under vacuum and heated to 70 °C for 1 hr for the AgCl to dissolve.

While the AgCl precursor dissolved, one 20 mL scintillation vial was loaded with degassed OLA 98% (1.5 mL) and a stir bar and brought inside a $N_2$ glovebox to prepare the $SnCl_4$ precursor solution. A micropipette was used to add $SnCl_4$ (18 µL, 0.15 mmol) to this vial. It is important to tilt the vial (~45°) and deliver the $SnCl_4$ onto a dry spot at the bottom of the vial to prevent traces of water in the OLA from hydrolyzing inside of the pipette tip and forming an inconvenient gel. For the synthesis of ATS@Zn NCs, another 20 mL scintillation vial was charged with $ZnBr_2$ (34 mg, 0.15 mmol) and degassed OLA 98% (1.5 mL) inside a $N_2$ glovebox. Both vials containing the $SnCl_4$ and $ZnBr_2$ precursors were taken outside of the glove box and then placed in a sand bath at ~70 °C for 30 min. When all metal precursors dissolved, two $(TMS)_2$-S solutions were prepared in different 20 mL scintillation vials inside the glove box. The first solution [1] contained toluene (1.5 mL) and $(TMS)_2$-S (26 µL/0.122 mmol), while the second [2] was comprised of toluene (1.5 mL) and $(TMS)_2$-S (98 µL/0.464 mmol). Each solution was loaded into a labelled 3 mL plastic syringe and taken out of the glovebox. This completed the precursor preparation.

The reaction temperature is set to 70 °C and the injection set up is assembled. A 3 mL plastic syringe is loaded with the tin solution (Sn-OLA) (a second syringe is loaded with the zinc precursor (Zn-OLA) for ATS@Zn NCs), both fully dissolved and pale yellow in colour. An additional 14/20 rubber septum is pierced with all four syringes as seen in Video 1. Then, the existing septum on the flask is removed under a positive nitrogen flow. This septum is quickly replaced by the one holding the syringes with the precursor solutions. The injection occurs at 70 °C in the order: 1) $(TMS)_2$-S [1], 2) Sn-OLA, and 3) $(TMS)_2$-S [2] with one second between injections. (a fourth injection 4) Zn-OLA is made for ATS@Zn NCs). The flask is removed from the heating mantle and rapidly cooled by blowing cold air. See attached videos for a full demonstration of the injection procedure.

To achieve larger sizes (>5 nm), the precursor injection was done at 80 °C and the reaction temperature was set to 95 °C. Aliquots for TEM analysis were taken after 10 minutes at 95 °C.

NCs are purified through a standard EtOH workup. Essentially, the crude solution (14 mL) is transferred and split into two 50 mL Falcon tubes and 14 mL of EtOH are added to each tube. The tubes are centrifuged at 4430 rfc for 3 minutes. The pale-yellow supernatant is discarded and the NCs are re-dispersed in 1 mL hexane. Then, ~700 µL of EtOH are added and the solution is

centrifuged at 4430 rfc for 30 seconds. The NC pellet is dispersed in 1 mL hexane for further experiments.

*Size-selective precipitation*: Using a micropipettor, 5 mL of crude solution were placed in a 50 mL Falcon tube. 7.5 mL of EtOH (99%) were added and the solution was centrifuged at 4430 rfc for 3 minutes. The pellet was re-dispersed in 500 µL of hexane and the supernatant was transferred into another Falcon tube to which 1.5 mL of EtOH were added. The solution was centrifuged at 4430 rfc for 2 minutes. Then, the pellet was re-dispersed in 500 µL hexane and the supernatant was collected into another Falcon tube and 1.5 mL EtOH were added, followed by centrifugation at 4430 rfc for 2 minutes. The pellet was re-dispersed in 500 µL hexane and the supernatant was transferred into another Falcon tube. EtOH was added until the 22.5 mL mark on the tube, followed by centrifugation at 4430 rfc for 2 minutes.

To isolate and stabilize the ATS cluster-like species, we added 1.5 mL of a 0.2 M $ZnBr_2$ in EtOH and 6 mL of pure EtOH instead of 7.5 mL of EtOH in the first purification step. All other steps were performed as described above. Cluster-like species are recovered in the third or fourth supernatant.

*Synthesis of reported ATS@Zn-2 NCs*: These NCs were synthesized following the procedure reported by Saha *et al*. at 160 °C for 2 hrs.[7]

*Purification procedure for HR-TEM*: NC samples were purified through a regular two-step purification with ethanol and hexane (See above). Then, 100 µL of the 1 mL stock solution were diluted in 200 µL hexane and this dispersion was placed in a vial containing 500 µL of a 0.075 M TBABr solution in DMF to create a bi-phasic system. 5 µL of butylamine were added into the hexane phase and the vial was closed and shaken for 10 seconds for the NCs to transfer from the hexane to the DMF phase. The hexane phase was removed with a pipette and 8-10 mL of toluene were added to precipitate the NCs *via* centrifugation for 30 seconds at 4430 rfc. The pellet was re-dispersed in 75 µL of DMF and toluene was carefully added dropwise until NC precipitation was observed (adding too much toluene will cause aggressive aggregation and the pellet will not be able to re-disperse for TEM analysis). The solution was centrifuged for 10 seconds at 4430 rfc, the supernatant was discarded, and the NCs were re-dispersed in 100 µL of DMF to create a stock solution. ~20 µL of this stock solution were diluted in 400 µL DMF to create a solution for drop casting onto TEM grids.

*Characterization:*

*Transmission Electron Microscopy (TEM):* BF TEM images were acquired using a Hitachi HT7700 microscope at 100 kV. 400-mesh copper grids (Pacific grid) were immersed in a dilute dispersion of NCs in hexane for 1 second and left to dry under air. The average size was determined over an average of 250-400 particles using the Fiji imaging-processing distribution of the ImageJ2 software. High-resolution measurements were acquired using either a Hitachi HF3300 equipped with a cold field emission electron gun, operated at 300 kV or a probe- corrected Thermo Fisher Spectra 300 S/TEM operated at 300 kV, employing a HAADF detector with a beam current of a few tens of picoamperes to limit beam damage to the sample. Compositional maps were acquired

using rapid raster scanning in Velox, with a probe current of ~150 pA. Elemental maps were produced after re-binning and local averaging within Velox.

*Steady-State Photoluminescence (PL):* Photoluminescence spectra taken with a home-built set-up at λ=450 nm excitation using a pen-diode (ThorLabs CPS450) at 2.5 mW. The emission set-up involved an off-axis parabolic collimating mirror to direct the emission from the sample to a reflective fibre-coupler (ThorLabs PC12FC-P01), which was then sent to an OceanOptics Flame spectrometer. The PLQY was measured by using a calibrated integrating sphere in a FS5 Spectrofluorimeter, Edinburgh Instruments ($\lambda_{ex}$=500 nm).

*Absorption spectroscopy (UV-Vis):* Optical absorption spectra were taken on an Agilent Cary 5000 UV/Vis spectrophotometer.

*X-Ray Diffraction:* XRD analysis was performed on a PANanalytical Empyrean X-ray diffractometer, equipped with a 1.8 kW Cu Kα ceramic anode and a PIXcel3D 2 × 2 area detector, operating at 45 kV and 40 mA. The samples were drop cast from hexane onto a silicon substrate and scanned for 6 to 24 hrs.

*Scanning Electron Microscopy Energy Dispersive Spectroscopy (SEM-EDS)*: Three drops of a 120 mg/mL stock NC dispersion in hexane were drop cast onto an amorphous silicon substrate and dried under ambient conditions overnight. SEM-EDS measurements were performed using a JEOL Dry SD30GV silicon-drift detector (SDD), with 30 $mm^2$ effective area.

*X-ray photoelectron spectroscopy (XPS):* A 40 mg/mL ATS dispersion in hexane was purified through five standard cycles of precipitation using EtOH and centrifugation, with re-dispersion in hexane at each step. For each of these washes, the pellet was re-dispersed in 1 mL hexane and 1 mL EtOH was added to induce precipitation of the NCs, followed by centrifugation at 3904 rfc for 1 min in each cycle. The clear supernatant was discarded after each centrifugation and the final pellet was dried under vacuum overnight.

Measurements were carried out using a Kratos Axis UltraDLD spectrometer (Kratos Analytical Ltd.) with a monochromated Al Kα X-ray source (hν = 1486.6 eV) operating at 20 mA and 15 kV. Each sample was grounded to the sample holder *via* copper tape to maximize its electrical conductivity. Wide-area scans were collected over an analysis area of 300 × 700 $\mu m^2$ at a photoelectron pass energy of 160 eV and energy step of 1 eV, while high-resolution spectra were collected at a photoelectron pass energy of 20 eV and an energy step of 0.1 eV. A take-off angle of 0° with respect to sample normal direction was used for all analyses. The differential electrical charging effects were neutralized. The spectra have been referenced to the adventitious carbon 1s peak at 284.8 eV. The spectra were analyzed with the CasaXPS software (Casa Software Ltd., version 2.3.24)[2] and the residual background was eliminated by the Shirley method.

*High-resolution powder X-ray diffraction and Pair distribution function analysis (PDF)*: High-resolution synchrotron X-ray diffraction and total scattering measurements were performed at beamline ID31 at the European Synchrotron Radiation Facility (ESRF). The sample powders were loaded into cylindrical slots (approx. 1 mm thickness) held between Kapton windows in a high-throughput sample holder. Each sample was measured in a transmission with an incident X-ray energy of 75.00 keV (λ = 0.1653 Å). Measured intensities were collected using a Pilatus CdTe 2M

detector (1679 × 1475 pixels, 172 × 172 μm$^2$ each) positioned with the incident beam in the corner of the detector. The sample-to-detector distance was approximately 1.5 m for the high-resolution measurements and 0.3 m for the total scattering measurement. Background measurements for the empty windows were measured and subtracted. NIST SRM 660b (LaB6) was used for geometry calibration performed with the software pyFAI followed by image integration including a flat-field, geometry, solid-angle, and polarization corrections.

High-resolution PXRD data were background subtracted. Preliminary PDF data were processed in an automated way using PDFgetX3 with Qmax = 25 Å$^{-1}$. Then the data were reprocessed using a Lorch modification function to suppress termination effects and contributions from high frequency noise. The small-angle scattering intensities were extrapolated from Qmin = 0.328 Å$^{-1}$ to 0. The compositions of the sample were taken into consideration to process de PDFs.

**Section 2**. Physical and Optical Characterization for ATS NCs

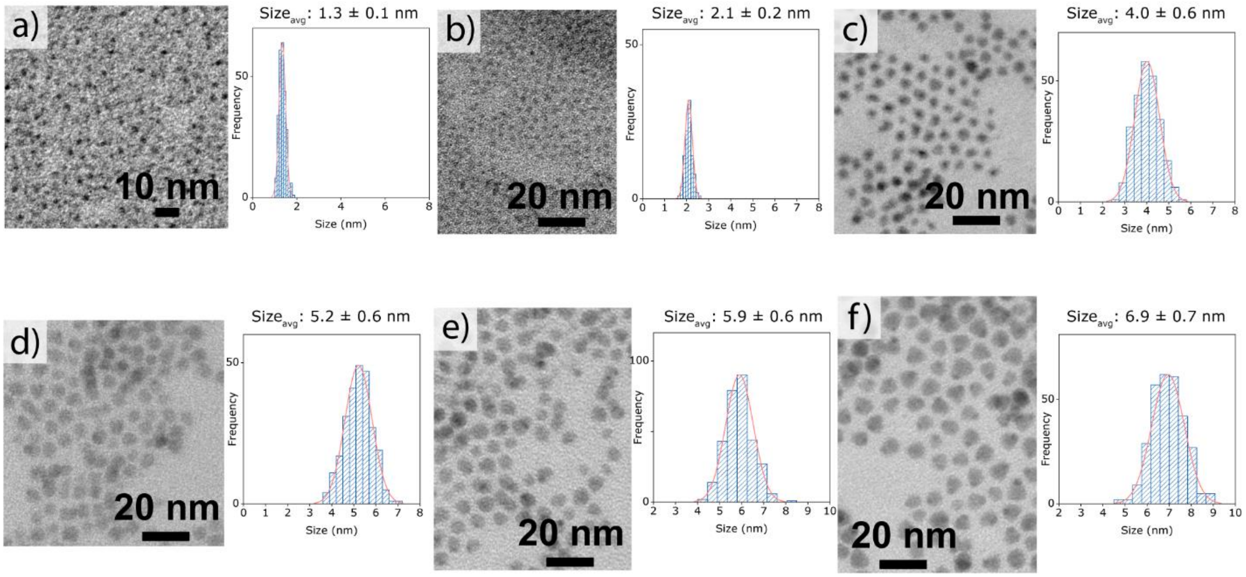


**Figure S 1**Error! Bookmark not defined.. BF TEM images and histograms showing size control for ATS (b to f) and ATS cluster-like species (a) NCs. The size of these NCs can be tuned in the 1.3 to 6.9 nm range.

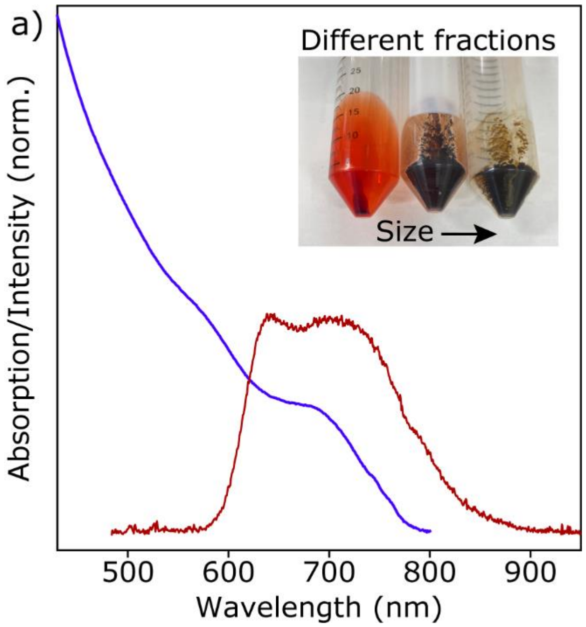


**Figure S 2**. Optical data UV-Vis (blue trace) and photoluminescence (red trace) for the whole product of a typical ATS NCs reaction. Various features can be observed in either spectra due to polydispersity.

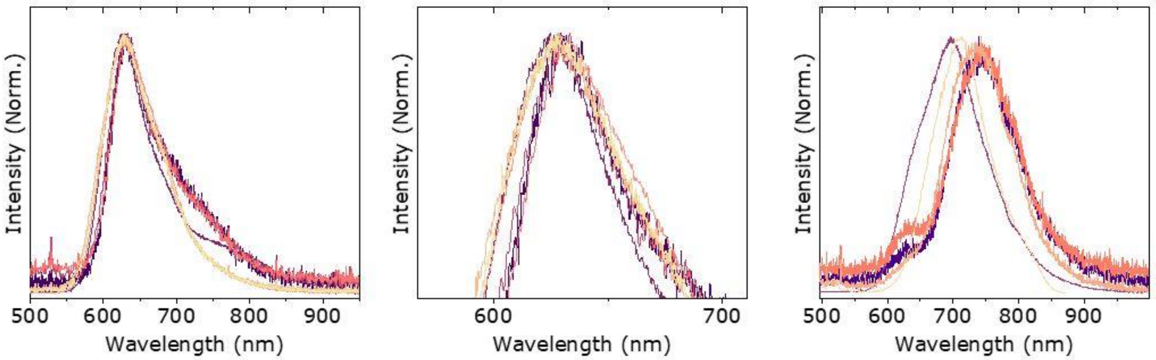


**Figure S 3.** a) PL spectra showing the reproducibility and consistency in emission (peak at ~630 nm) for ATS cluster-like species over 7 syntheses performed in different days and laboratories (The University of Toronto and the Istituto Italiano di Tecnologia). b) Peak zoomed in of (a), and c) PL spectra for larger ATS NCs shows emission variability.

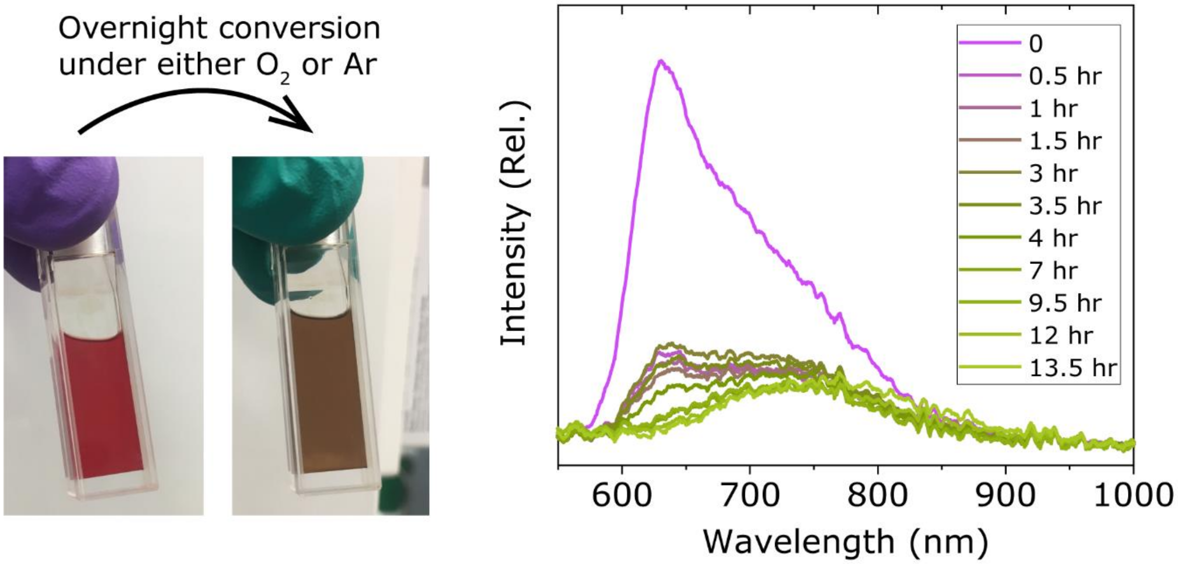


**Figure S 4**. Cluster-like ATS species with emission peak at ~630 nm converts to larger ATS NCs overnight under constant 450 nm laser irradiation (~2 mW at the focal point). While a continue laser was used here to enable the acquisition of the time-series of emission spectra, we do not consider that laser excitation is necessary for the conversion. This experiment was performed under an argon atmosphere by purging the solution in a sealed cuvette for 2 minutes and leaving it at a positive argon pressure *via* a ballon.

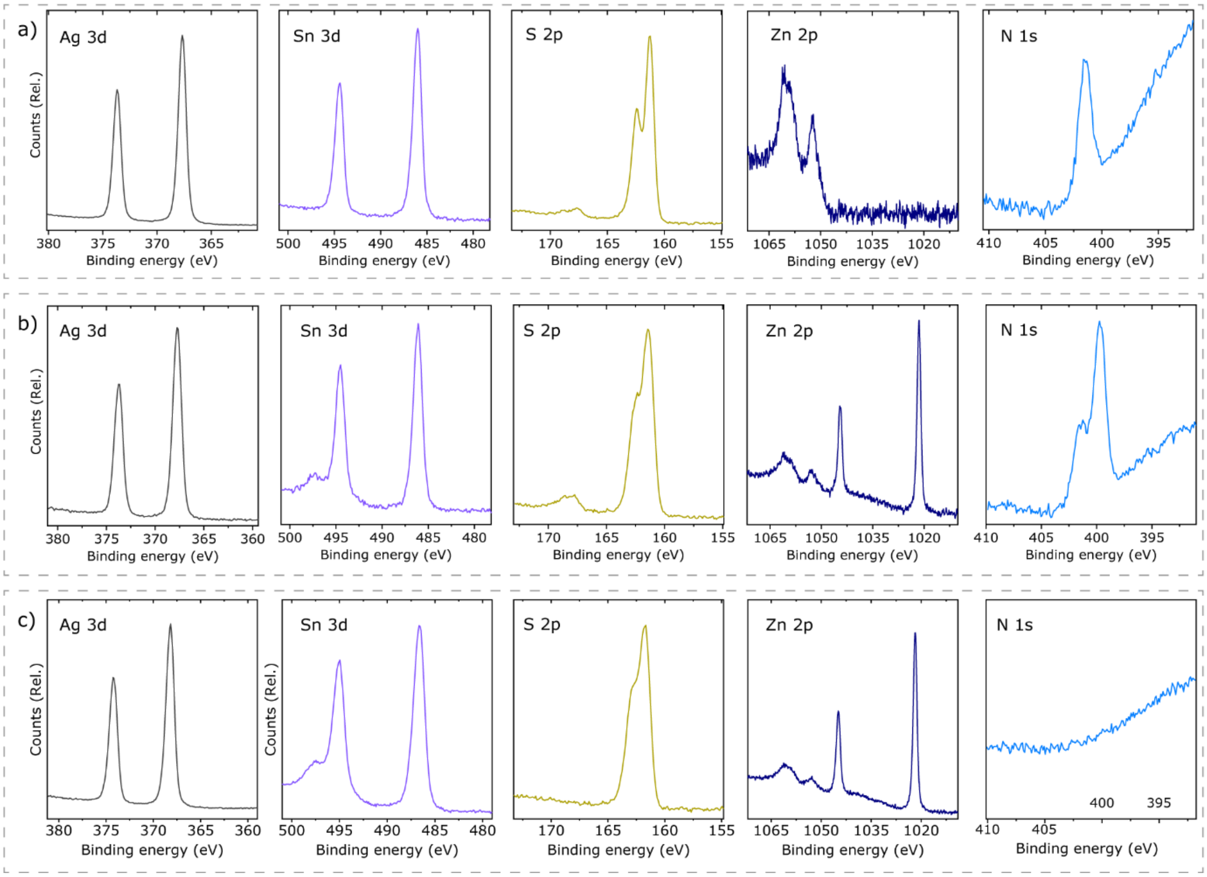


**Figure S 5**. X-ray photoelectron spectroscopy data for a) ATS, b) ATS@Zn-1, and c) ATS@Zn-2 NC samples of average size 6.0 nm. The most relevant observations in this set of data concerns elements Sn, S, and N (See XPS discussion below).

Sn: Quantitative analysis suggests that tin is present as Sn (IV) in both systems. However, determination of the oxidation state of Sn is not straightforward, as Sn (II) and Sn (IV) signals often overlap. Typically, the analysis requires the combination of XPS and Auger data to obtain a Wagner plot.[3] Such analysis on ATS and ATS@Zn samples suggests that Sn is in a 2+, contrary to our quantitative analysis *via* XPS, which might indicate that some Sn (II) is present at the surface where it is more prone to redox reactions. Although the behaviour of Sn in terms of oxidation states in the lattice is interesting, it is out of the scope of this study.

S: The doublet fitting requires three different components, which might suggest that there is some sulfur oxidation at the surface (*e.g.,* sulfone).

N: A second nitrogen component appears when Zn is present in the reaction. This observation indicates that there are two distinct coordination environments for OLA on the surface. Similarly, it aligns with our previous observations into the surface chemistry of these class of NCs where we observed weaker OLA-metal sites as well as tightly bound $Zn(OLA)_x$ ligands on surface sulfur atoms.[4]

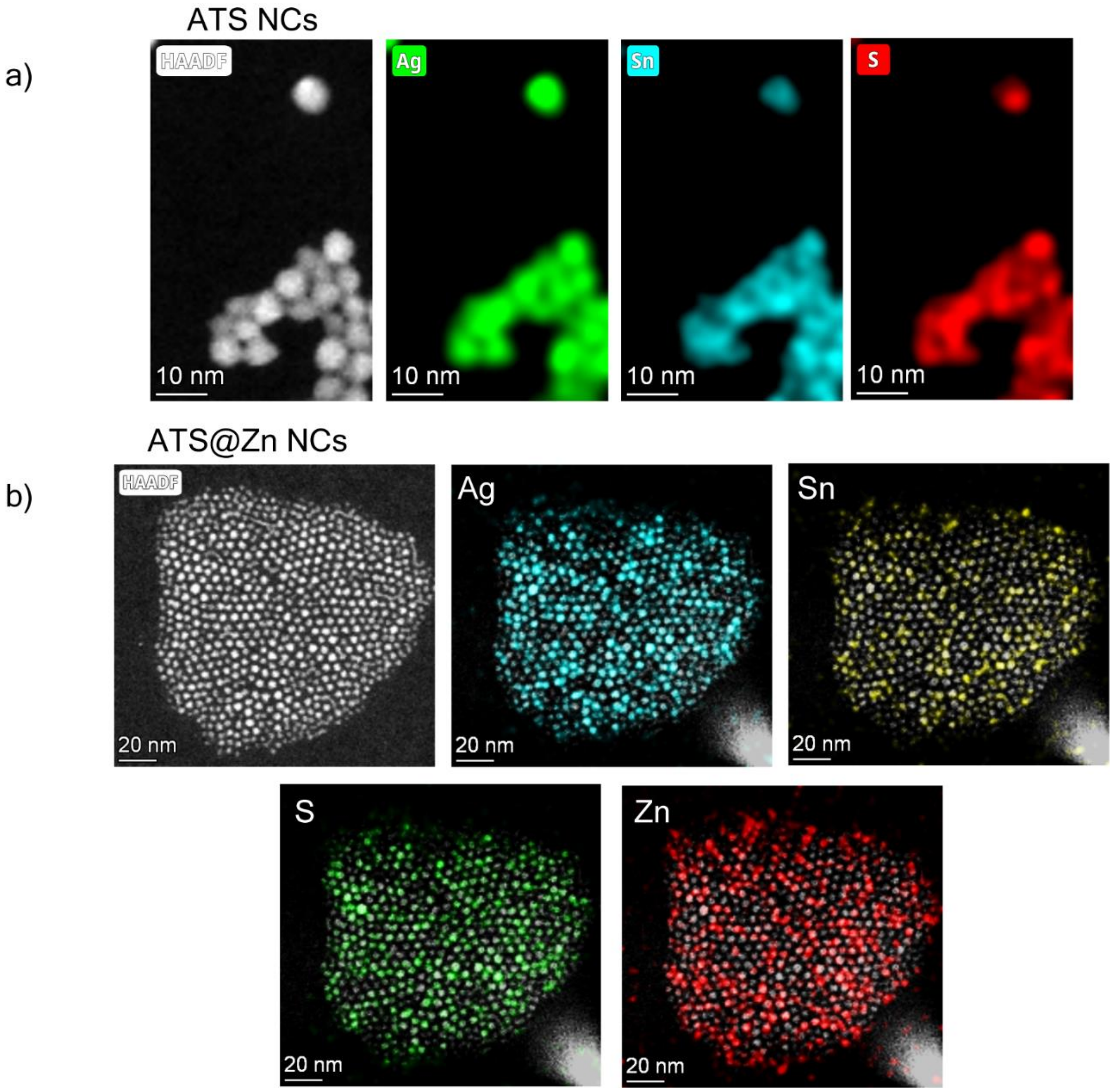


**Figure S 6**. Elemental maps via STEM-EDX of a) ATS and b) ATS@Zn-1 NCs.

**Table S1**. Stoichiometry data acquired through different elemental analysis techniques.

| Sample | Technique | Ag | Sn | S | Zn |
|---|---|---|---|---|---|
| **ATS** | XPS | 3.3 | 1.0 | 3.6 | - |
| | STEM-EDX | 4.0 | 1.0 | 3.2 | - |
| **ATS@Zn-1** | XPS | 2.2 | 1.0 | 3.9 | 0.7 |
| | STEM-EDX | 3.9 | 1.0 | 4.8 | 2.1 |
| | SEM-EDS | 2.3 | 1.0 | 4.1 | 1.4 |
| **ATS@Zn-2** | XPS | 2.6 | 1.0 | 3.4 | 0.8 |

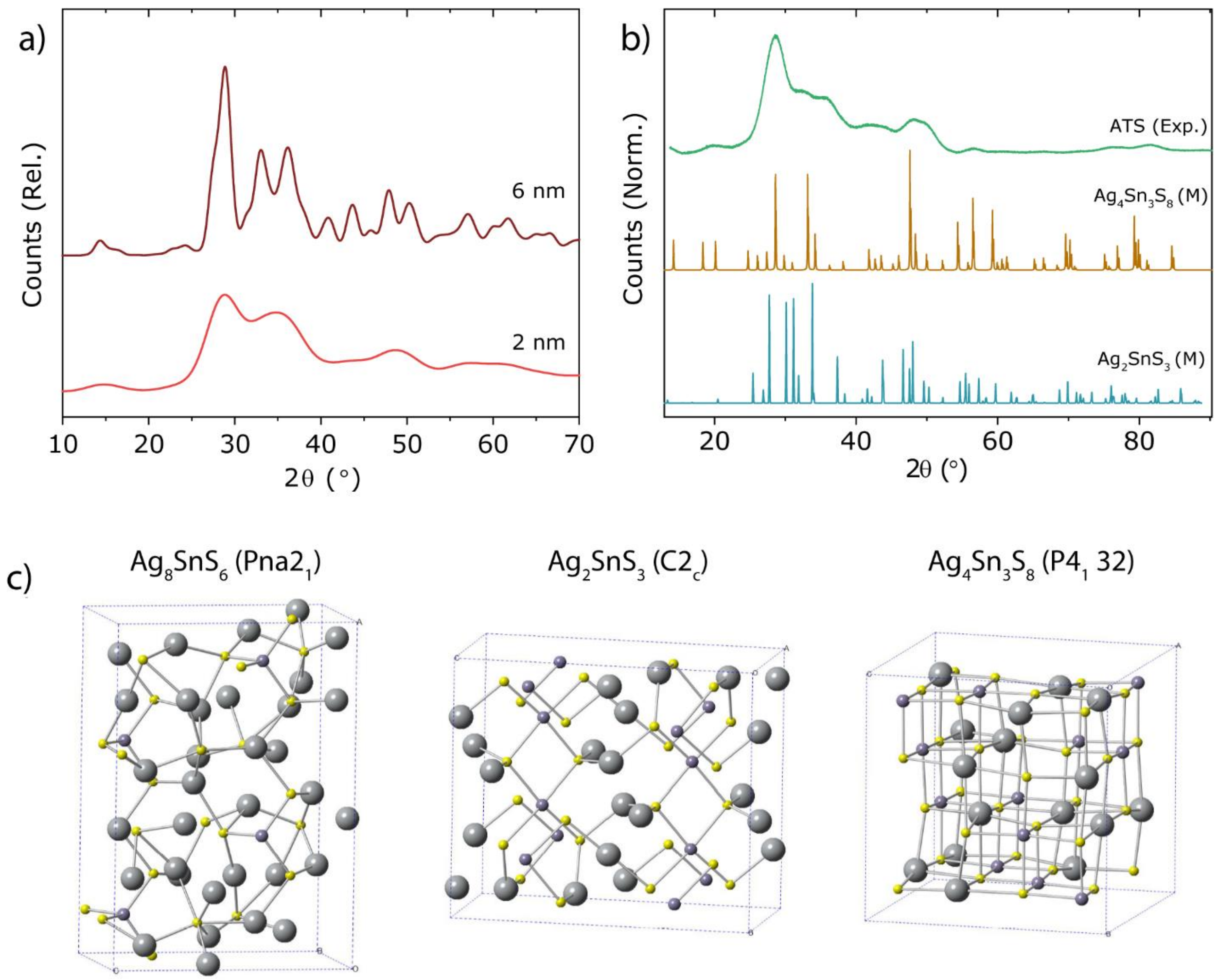


**Figure S 7**. Crystallographic data for ATS and ATS@Zn-1 NCs. a) Simulated PXRD diffractograms, using Crystal Diffract, for Canfieldite with crystal sizes of 2 and 6 nm. b) High-resolution PXRD of ATS NCs (~6.5 nm, green trace) compared to two monoclinic phases in the Ag-Sn-S system, PDF 00-038-0245 ($Ag_4Sn_3S_8$) and PDF 00-039-0140 ($Ag_2SnS_3$). c) Unit cell models for orthorhombic Canfieldite, monoclinic $Ag_2SnS_3$, and monoclinic $Ag_4Sn_3S_8$.

### *Rietveld refinement details (Figures 1c and d)*

Simple Rietveld refinements were performed to both the cubic and orthorhombic models to the background subtracted diffraction patterns to see how plausible the indexing of either model is to the observed diffraction features.

In the fits, the lattice parameters were kept constant since the diffraction features are extremely broadened, resulting in a very low sensitivity to lattice parameter values, and to avoid any unrealistic distortions away from the published structure models. Only a scale factor, Gaussian crystallite size broadening term, and the Stephens model using either cubic or orthorhombic setup to allow for anisotropic broadening of the reflections from the crystal models, were refined to fit the peak shapes. The background was described using a Chebychev polynomial of 7th order, and constrained between both refinements so that the resulting fits could be sensibly compared.

**Section 3**. Supporting PXRD, TEM, and electron diffraction data

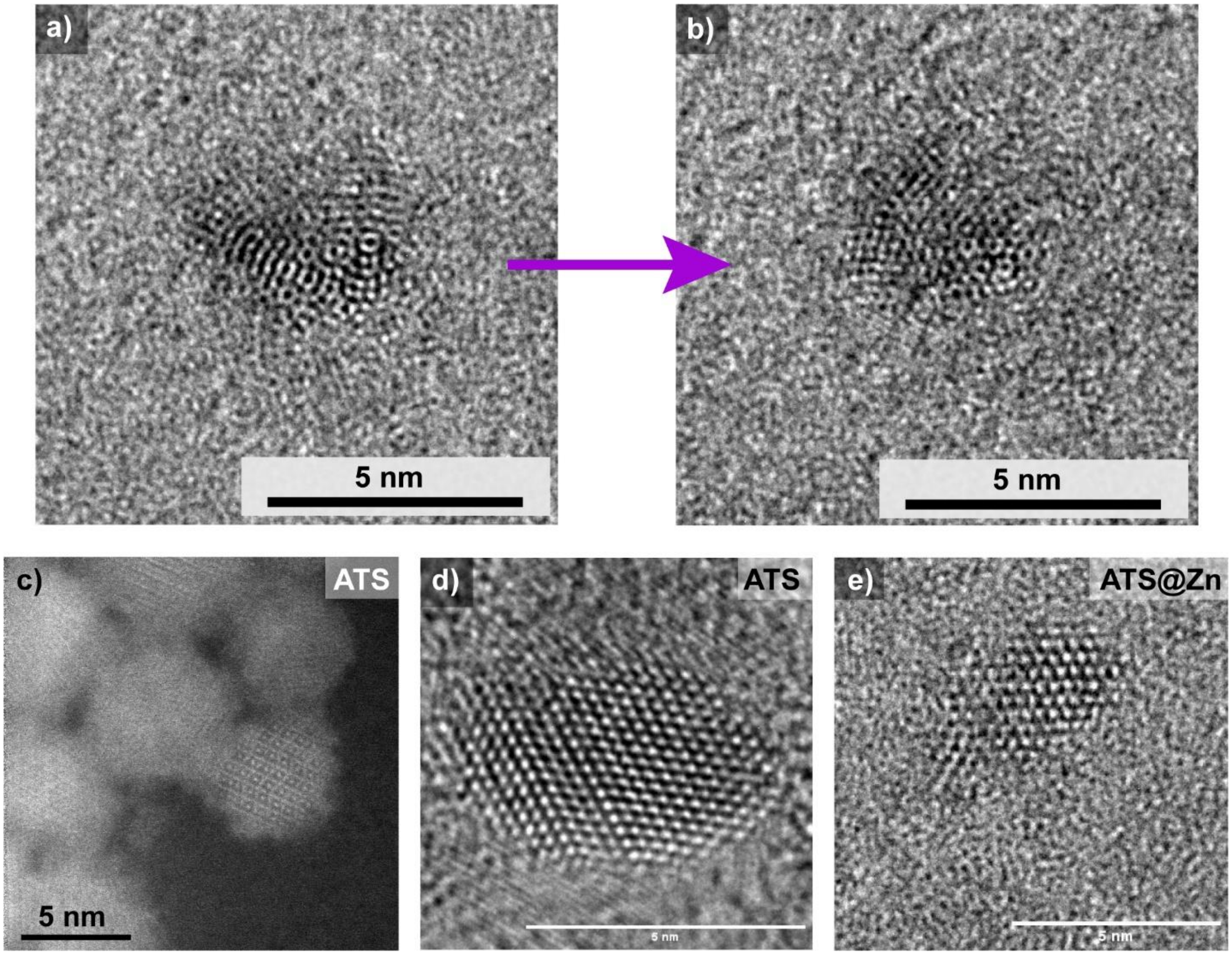


**Figure S 8**. HR-(S)TEM images for ATS and ATS@Zn-1 NCs. a and b) show the coalescence of polycrystalline NCs into single domains under the electron beam. c and d) Single-phase ~5 nm ATS NC displaying stacking faults. e) Single-phase anisotropic ATS@Zn-1 NC.

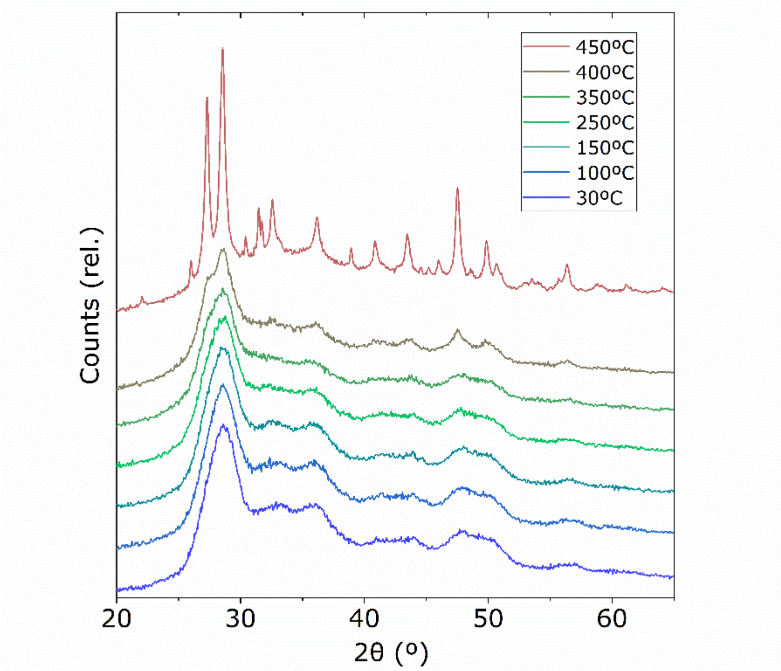


**Figure S 9**. Temperature-dependent PXRD measurements for ATS NCs (~5 nm) on an amorphous silicon substrate. The measurements show little change in the pattern until ~400 °C in which more reflections can be recognized due to crystallite domain growth.

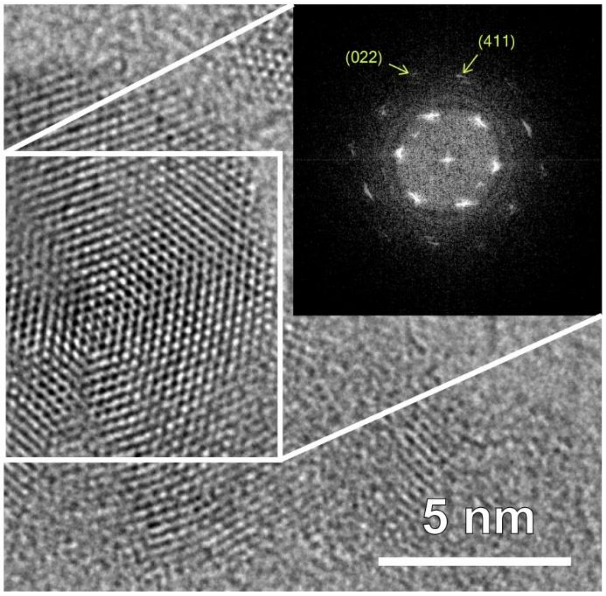


**Figure S 10**. HR-TEM image and corresponding FFT pattern for an ATS NC. The planes (022) and (411) correlate with d-spacing values 0.311 and 0.301 nm matching the orthorhombic phase of canfieldite. (*c.f.* Figure 3)

**Section 4**. PDF analysis.

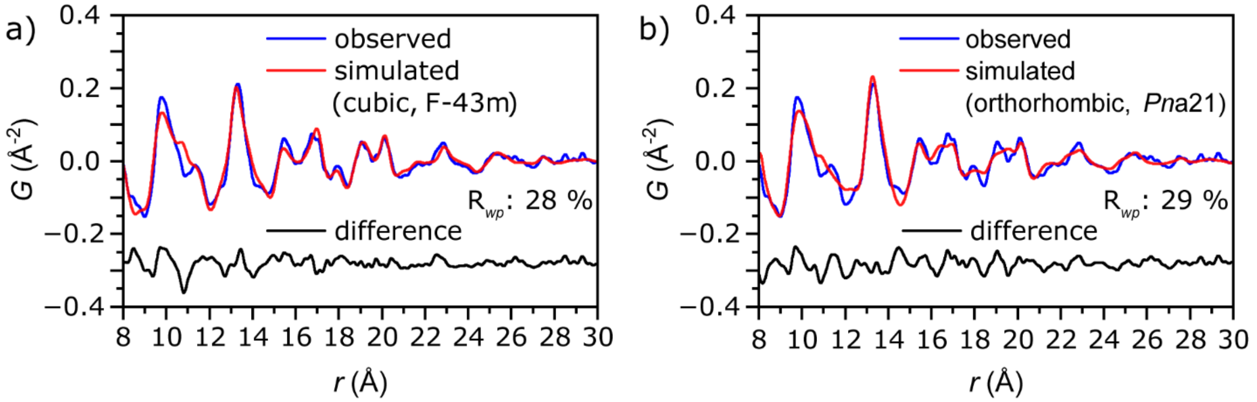


**Figure S 11**. Results of real-space fits of the unmodified cubic (a) and orthorhombic (b) structures to the intermediate range structure observed by the measured PDF (fitted with PDFgui).

### *Modelling information*

The models were developed using the TOPAS v7 software using the parameters described in Table S2. Further parameters included lattice parameters, scale factor, isotropic ADPs for Ag, S, and Sn, an intermolecular ADP for $SnS_4$ tetrahedra, and a separate correlated motion parameter delta2 for Ag atoms to model sharpened distance distribution between it and nearest neighbor S or Ag atoms.

**Table S2**: Summary of cubic and orthorhombic models and their constrain parameters

| Model / Constrain | Pseudo cubic | Orthorhombic | Pseudo-orthorhombic (I) | Pseudo-orthorhombic (II) |
|---|---|---|---|---|
| Lattice | a=b=c | a≠b≠c | a≠b≠c | a≠b≠c |
| Symmetry | P1 | Pna21 | P1 | P1 |
| Ag | refine independently of cubic symmetry | refine following orthorhombic symmetry | refine following orthorhombic symmetry | refine independently of orthorhombic symmetry |
| $SnS_4$ | rigid body / fixed position | rigid body / fixed position | rigid body / allowed to rotate | rigid body / allowed to rotate |
| S | allowed to rotate around site | fixed position | allowed to rotate around site | allowed to rotate around site |

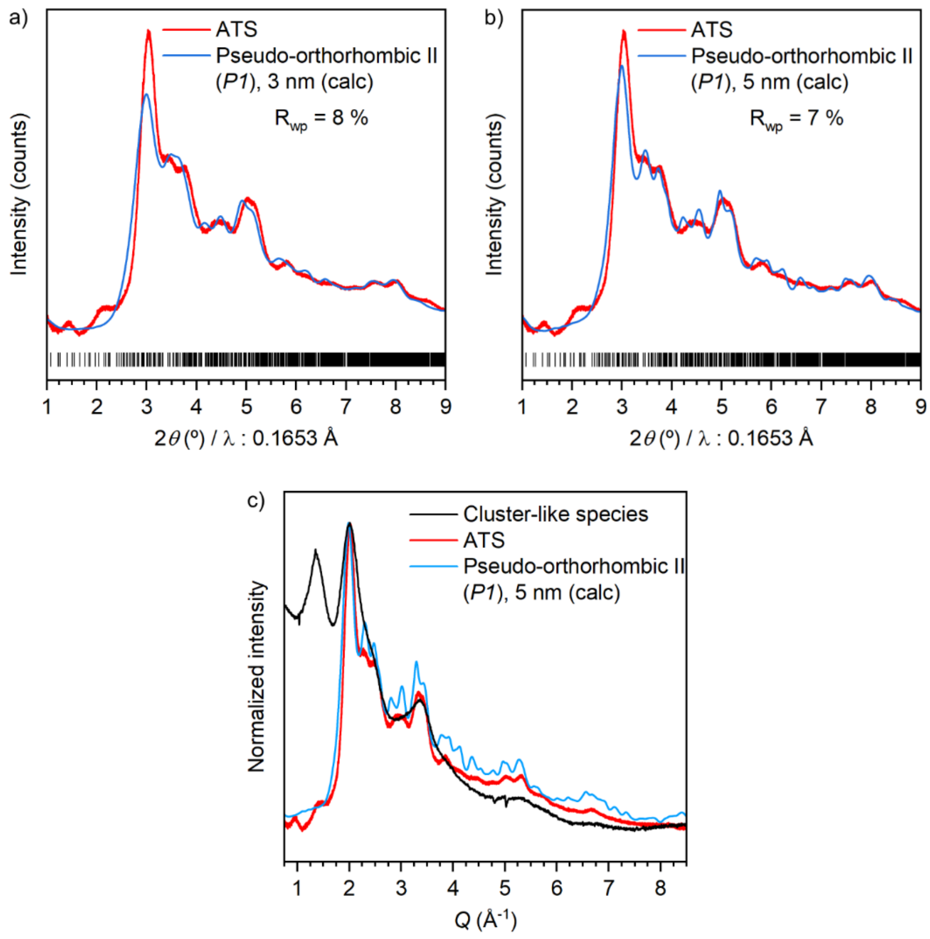


**Figure S 12**. Simulated diffraction patterns of the Pseudo-Orthorhombic (P1) structure model obtained after refinement through PDF analysis, (a) considering 3 (Rw = 8%) or (b) 5 nm (Rw = 7%) particle size, and (c) comparison with diffraction pattern of ATS and cluster-like species in Q-space.

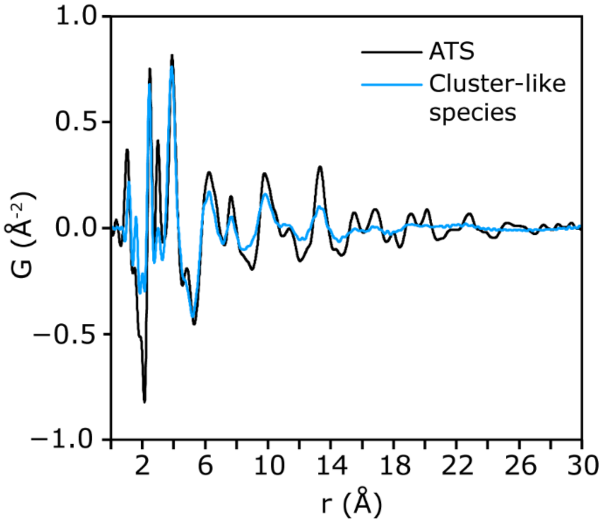


**Figure S 13**. PDF data comparing ATS to cluster-like species. There is a high similarity in the local structure (0-5Å). Thus, cluster-like species are compatible with a pseudo-orthorhombic model.

**Section 5**. Physical and optical characterization for ATS@Zn NCs

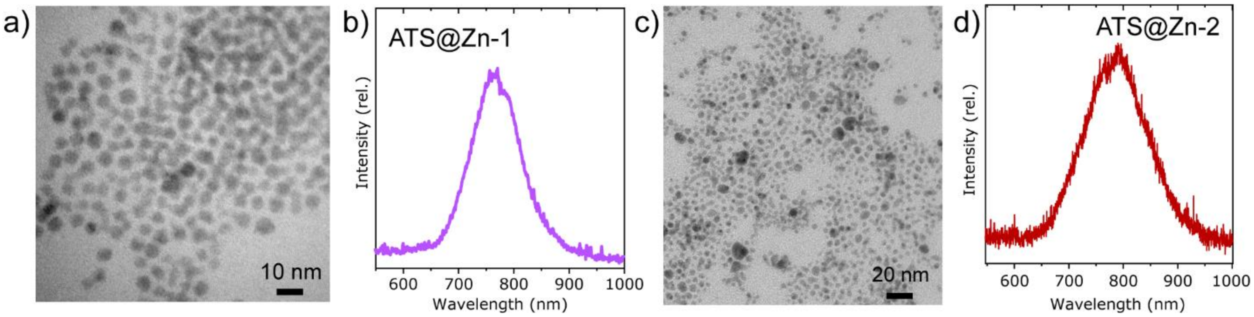


**Figure S 14**. BF TEM and photoluminescence data for ATS@Zn-1 (a and b) and ATS@Zn-2 NCs (c and d).

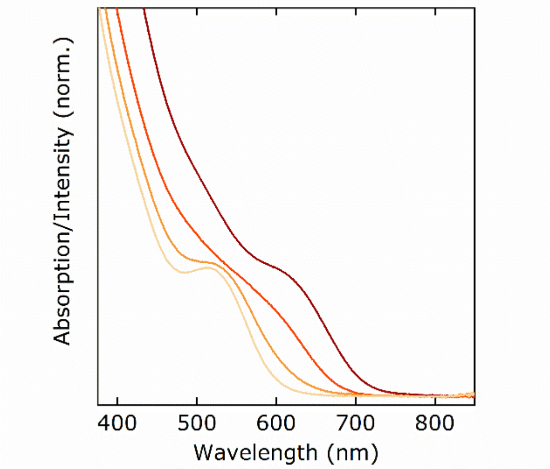


**Figure S 15**. Optical absorption spectra of aliquots taken from an ATS@Zn-1 reaction at different times after precursor injection (from 10 s to 30 min.)

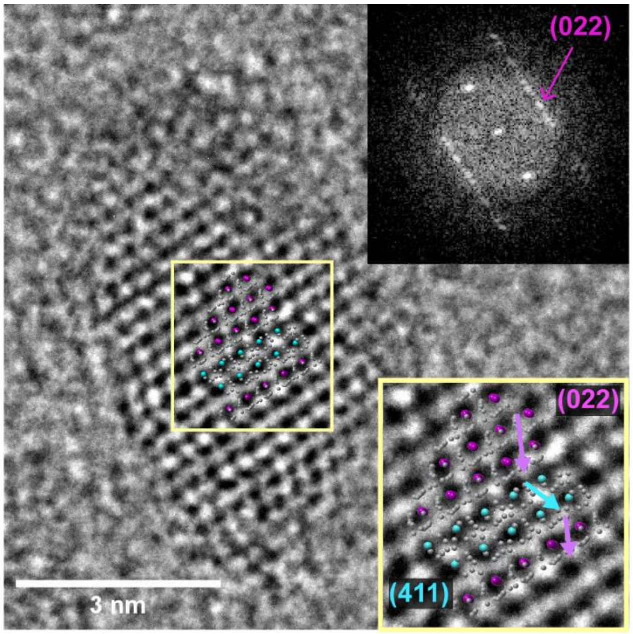


**Figure S 16**. HR-TEM image and corresponding FFT pattern for an ATS@Zn-1 NC displaying a stacking fault. Sn and Ag atoms are overlaid in pink/cyan and silver, respectively. The lattice propagates through the (022) plane with d-spacing of 0.311 nm as confirmed *via* diffraction (see inset). Then, the lattice faults and starts to propagate on the (411) plane for one lattice spacing (0.301 nm) until it returns to the (022) plane.

*Associated discussion for Figure S15*

We identified several ATS NCs with stacking faults. To get insight into the preferred planes in which the faults were originating, we calculated the d-spacing through these faults (*i.e.,* 3.11 and 3.01 Å). These values are consistent with planes (022) and (411) for orthorhombic canfieldite and constitute the major reflections in the XRD pattern, suggesting that ATS NCs might have a tendency to growth through those planes. This preferred orientation growth behavior would be expected in such structures with anisotropic lattices. Overall, these results support our PDF analysis in confirming that our NCs have a canfieldite phase.

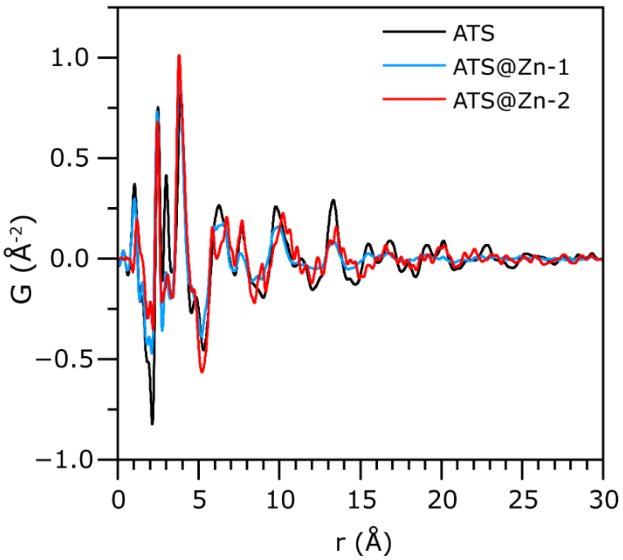


**Figure S 17**. Extended range PDF data for ATS, ATS@Zn-1, and ATS@Zn-2 showing the similarity in the local structure to a canfieldite-like phase. A subset of this data is presented as Figure 5d.

## References


1. Yarur Villanueva, F.; Hasham, M.; Green, P. B.; Imperiale, C. J.; Rahman, S.; Burns, D. C.; Wilson, M. W. B., A Stepwise Reaction Achieves Ultrasmall $Ag_2ZnSnS_4$ Nanocrystals. *ACS Nano* **2024,** *18* (52), 35182-35201.
2. Fairley, N. F., V.; Richard□Plouet, M.; Guillot-Deudon, C.; Walton, J.; Smith, E.; Flahaut, D.; Greiner, M.; Biesinger, M.; Tougaard, S.; Morgan, D.; Baltrusaitis, J. , Systematic and Collaborative Approach to Problem Solving Using X-Ray Photoelectron Spectroscopy. *Appl. Surf. Sci.* **2021,** *5*, 100112.
3. Wieczorek, A.; Lai, H.; Pious, J.; Fu, F.; Siol, S., Resolving Oxidation States and X–site Composition of Sn Perovskites through Auger Parameter Analysis in XPS. *Advanced Materials Interfaces* **2023,** *10* (7), 2370024.
4. Villanueva, F. Y. *Understanding and Controlling the Formation Mechanism and Surface Chemistry of Lead-Free Quaternary Semiconductor Nanocrystals*. PhD thesis, University of Toronto, ProQuest, September 2024.
